# A digital assay for programmed death-ligand 1 (22C3) quantification combined with immune cell recognition algorithms in non-small cell lung cancer

**DOI:** 10.1038/s41598-022-12697-1

**Published:** 2022-06-13

**Authors:** Will Paces, Elliott Ergon, Elizabeth Bueche, G. Dave Young, Vitria Adisetiyo, Cris Luengo, Meredith James, Charles Caldwell, Dannah Miller, Morgan Wambaugh, Geoffrey Metcalf, Roberto Gianani

**Affiliations:** 1 Flagship Biosciences, Inc., Broomfield, CO USA; 2 Flagship Biosciences, Inc., 11800 Ridge Pkwy, Suite 450, Broomfield, CO 80021 USA

**Keywords:** Machine learning, Cancer

## Abstract

PD-L1 (22C3) checkpoint inhibitor therapy represents a mainstay of modern cancer immunotherapy for non-small cell lung cancer (NSCLC). In vitro diagnostic (IVD) PD-L1 antibody staining is widely used to predict clinical intervention efficacy. However, pathologist interpretation of this assay is cumbersome and variable, resulting in poor positive predictive value concerning patient therapy response. To address this, we developed a digital assay (DA) termed Tissue Insight (TI) 22C3 NSCLC, for the quantification of PD-L1 in NSCLC tissues, including digital recognition of macrophages and lymphocytes. We completed clinical validation of this digital image analysis solution in 66 NSCLC patient samples, followed by concordance studies (comparison of PD-L1 manual and digital scores) in an additional 99 patient samples. We then combined this DA with three distinct immune cell recognition algorithms for detecting tissue macrophages, alveolar macrophages, and lymphocytes to aid in sample interpretation. Our PD-L1 (22C3) DA was successfully validated and had a scoring agreement (digital to manual) higher than the inter-pathologist scoring. Furthermore, the number of algorithm-identified immune cells showed significant correlation when compared with those identified by immunohistochemistry in serial sections stained by double immunofluorescence. Here, we demonstrated that TI 22C3 NSCLC DA yields comparable results to pathologist interpretation while eliminating the intra- and inter-pathologist variability associated with manual scoring while providing characterization of the immune microenvironment, which can aid in clinical treatment decisions.

## Introduction

Determination of programmed death-ligand 1 (PD-L1) expression in tumors by immunohistochemistry (IHC) is often used to predict patient response to checkpoint inhibitor therapies. In particular, the Dako PD-L1 (22C3) antibody is a widely used companion diagnostic to the monoclonal antibody drug pembrolizumab in non-small cell lung cancer (NSCLC)^1,2^. However, for the practicing pathologist, interpretation of the PD-L1 (22C3) assay is cumbersome and time consuming. This is compounded by the high intra- and inter-pathologist variability of manual scoring which can potentially lack accuracy in less experienced readers^3–6^. Furthermore, the positive predictive value of the current 22C3 PD-L1 staining paradigm is less than ideal with many patients failing to respond to checkpoint inhibitor therapy despite harboring designated PD-L1 positive tumors^1,2,7^. In addition, these traditional manual reads may not fully capture the complex interaction between tumor cells and the immune microenvironment. There is growing literature on the significance of macrophage and lymphocyte PD-L1 positivity for predicting treatment response, highlighting the need to differentiate PD-L1 positivity in tumor cells versus tumor associated immune cells (i.e., macrophages and lymphocytes in the tumor compartment)^8,9^. Kumagai et al. showed higher PD-1 expression on CD8^+^ lymphocytes in NSCLC can be considered a predictive biomarker for therapeutic antibody effectiveness^10^. This need is further underscored by the tendency of manual and digital reads to confuse alveolar macrophages as tumor cells^11^. In order to address these challenges, we developed a novel digital assay known as Tissue Insight for the quantification of PD-L1 (22C3) in tumor cells and macrophages of NSCLC (TI 22C3 NSCLC DA).

In this study, we successfully assessed the performance of TI 22C3 NSCLC DA. First, by clinical validation of the assay for analytical sensitivity, specificity, accuracy, and precision, according to applicable Clinical Laboratory Improvement Amendments (CLIA) standards (> 90% for all validation parameters). Secondly, through the evaluation of concordance testing of the validated assay with manual pathology. Finally, we show the development of additional algorithms which digitally recognize macrophages (tissue and alveolar) and lymphocytes based on the morphological features as determined by image analysis (IA) training. Applying these algorithms to NSCLC digital slides analyzed by TI 22C3 NSCLC DA was done to determine the possibility of adding macrophage and lymphocyte recognition algorithms to the TI 22C3 NSCLC DA without altering (and potentially improving) the accuracy of the Tumor Proportion Score (TPS) determination. All three immune cell recognition algorithms exhibited a significant correlation (r > 0.80, p < 0.001) between IA solution predicted macrophage and lymphocyte cell counts and the double immunofluorescence CD163/CD68 and CD3/CD20 identified counts respectively. In sum, we have shown this validated digital assay can aid in the cumbersome manual interpretation of patient treatment, while eliminating pathologist variability associated with manual scoring. Furthermore, the assay can be successfully combined with immune cell recognition algorithms providing additional data on the number of immune cells within the tumor and stoma compartment as well as their PD-L1 status.

## Results

### Part 1: clinical validation of TI 22C3 NSCLC DA

#### Staining assessment

IHC staining was evaluated by the pathologist for intensity and distribution in appropriate target cells and in subcellular localizations. As expected, all 66 samples in the validation study cohort stained with the anti-PD-L1 (22C3) antibody exhibited absent to strong membrane and cytoplasm staining of immune cells (i.e., T cells, macrophages) and absent to strong membrane staining of certain tumors. There was an absence of nonspecific background staining for all sections stained with the negative isotype control reagent. Background staining exhibited in all samples was very low to absent.

#### Reportable range

The reportable range of this assay is 0% to 100% on the TPS scale. The lowest and highest TPS scores reported for the NSCLC indication were all within the assay’s expected limits of detection: 0% and 89.87% for the digital TPS scoring and 0% and 100% for the manual pathology TPS scoring.

#### Validation parameters assessment

The clinical validation study demonstrated that the digital PD-L1 (22C3) assay yields high analytical sensitivity, specificity, accuracy, and precision in the determination of the PD-L1 score as > 90% of the tissue cohort met the predetermined CLIA passing criteria for all validation parameters (Table 1).

**Table 1:** Validation parameters of the PD-L1 digital assay.

Validation parameter	Parameter definition	Pass/fail
Analytical Specificity	The assay demonstrates acceptable staining as identified by specific cell type and subcellular localization of staining	Pass
Analytical Sensitivity	The assay demonstrates acceptable target cell staining at various intensities and acceptable background staining. Background staining must be < 1 + staining intensity for acceptability	Pass
Accuracy	The assay demonstrates acceptable concordance with an orthogonal measurement of the assay’s determinant	Pass
Precision	The assay demonstrates acceptably reproducible staining over 3 runs	Pass

Of the 66 NSCLC specimens evaluated, 97% (64/66) of the tissue cohort passed the specificity acceptance criteria by exhibiting appropriate cell identification (i.e., ability of the algorithm to accurately identify cells when present, with ≤ 10% false negative rate for staining classification [i.e., ability of the algorithm to accurately classify positive cells as positive]). Of the specimens evaluated, 100% (66/66) of the tissue cohort passed the sensitivity acceptance criteria by exhibiting acceptable target cell staining and background staining with the background < 1 + staining intensity. Ninety-one percent (60/66) of the tissue cohort passed the accuracy acceptance criteria by exhibiting concordance in non-treatment (TPS < 1%) vs. treatment (TPS ≥ 1%) according to the digital and manual pathology TPS evaluation. Lastly, 100% of the tissue cohort passed the precision acceptance criteria (by exhibiting concordance in non-treatment (TPS < 1%) vs. treatment (TPS ≥ 1%) binning according to the digital TPS evaluation of the same samples stained on different days in the same lab (within lab precision: Day 2 vs. Day 3) and between different labs (between lab precision: Day 1 vs. Day 2, Day 1 vs. Day 3).

### Part 2: concordance testing

#### Staining assessment and reportable range

The 66 NSCLC samples used in the clinical validation were combined with an additional 99 patient samples to conduct concordance testing to evaluate the benefits of TI 22C3 NSCLC DA. Upon qualitative evaluation, all samples in the concordance testing cohort stained appropriately for PD-L1 (22C3), with very low to absent nonspecific background staining. The lowest and highest TPS scores reported for the NSCLC indication were all within the assay’s expected 0–100% limits of detection: 0.09% and 90.297% for the digital TPS scoring and 0% and 100% for the manual pathology TPS scoring (Pathologist 1: 0–95%, Pathologists 2 and 3: 0–100%).

#### Digital-to-manual vs. manual-to-manual concordance

Variability among PD-L1 scoring was evaluated in all patient samples by first evaluating our novel TI 22C3 NSCLC DA compared to pathologist’s manual evaluation of PD-L1 scoring in a digital-to-manual assessment. Secondly, pathologist variability was assessed through a manual-to-manual concordance test. The digital-to-manual PD-L1 (22C3) scoring agreement for the first scoring between the TPS obtained from the digital assay and from each pathologist was higher and statistically significant (Table S1). This was also comparable to the manual-to-manual concordance observed between the three different pathologists (Table S1).

#### Intra-pathologist vs. intra-digital concordance

When comparing the pathologist’s agreement in PD-L1 scoring, the intraclass correlation coefficient (ICC) analysis of the intra-pathologist across the three repeated scoring demonstrated excellent reliability in scoring for each pathologist (Table S2). Specifically, ≥ 90% of the variance in the manual pathology TPS endpoint was attributable to inter-sample variance and residual error, as opposed to the variance resulting from repeated scoring. While the intra-pathologist concordance was high across the three repeated scoring, the intra-digital TPS concordance still outperformed the manual concordance by demonstrating zero variance between the three repeated scoring (Table S2).

### Part 3: addition of macrophage and lymphocyte IA-based solutions

#### Staining assessment

To enhance the benefits of the validated TI 22C3 DA, we developed additional algorithms that can be added to the assay and provide additional insight into the patient’s sample. To assess the algorithms, we conducted traditional staining to compare our algorithms. Immunohistochemistry (IHC) PD-L1 (22C3) staining, IF CD163/CD68 staining, and IF CD20/CD3 staining were all evaluated by the same pathologist for intensity and distribution in appropriate target cells and in subcellular localization. All 99 samples in the sample cohort stained appropriately with the anti-PD-L1 (22C3) antibody. The pathologist confirmed appropriate CD163/CD68 and CD20/CD3 staining was observed in the IF-stained samples. Background staining in all samples was very low to absent. Based on this qualitative pathology assessment, 99 samples (consisting of 26 whole tissue samples and 73 tissue microarray [TMA] cores) were analyzed for the macrophage and lymphocyte IA-based solutions.

#### Algorithm performance assessment

**Figure 1:**
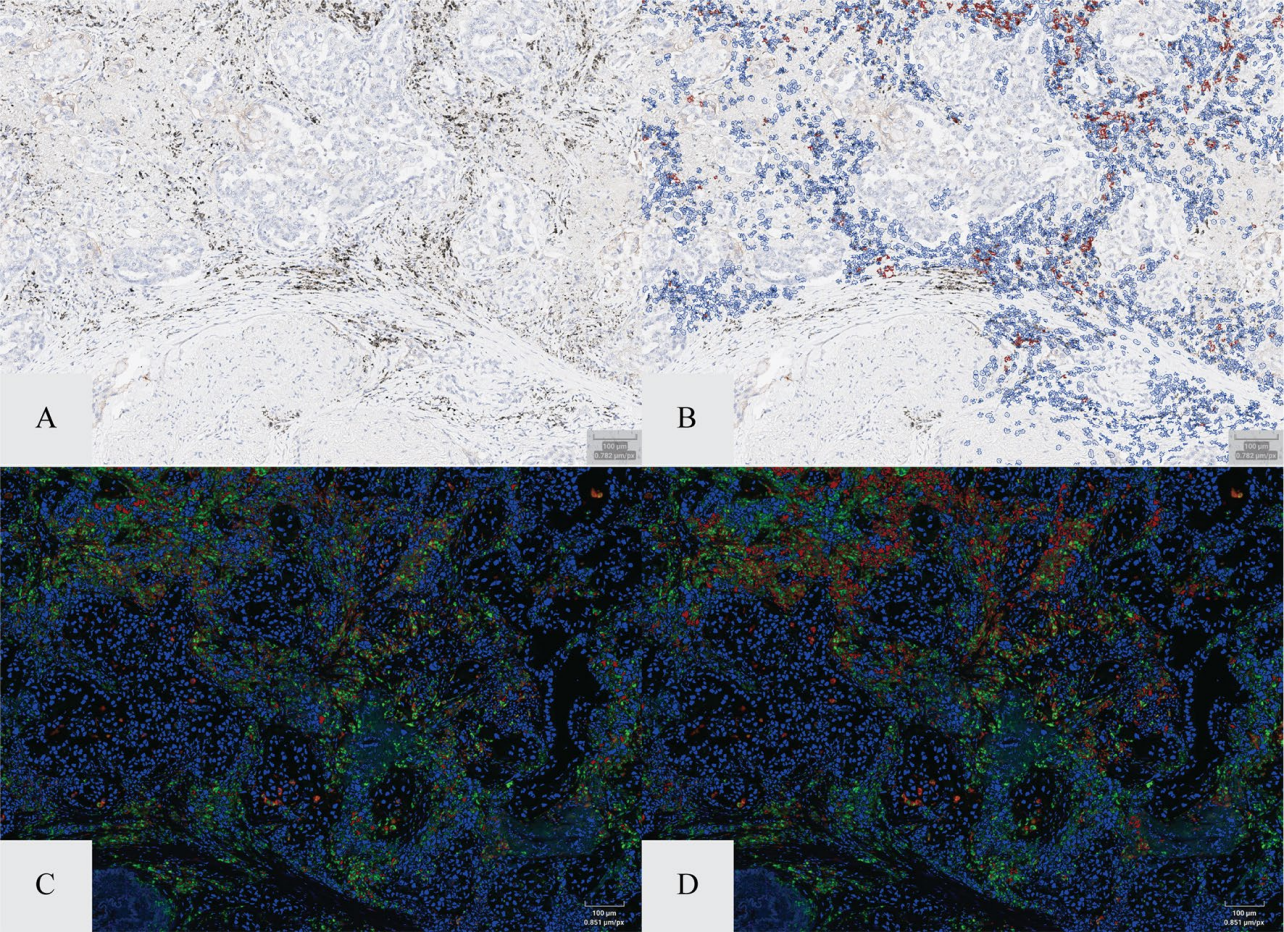
IA vs. double immunofluorescence of macrophage identification. Panel (**A**) and (**B**) show PD-L1 stained tumor field of view with and with out the macrophage algorithm labeling (red = PD-L1 positive; blue = PD-L1 negative). Panel (**C**) and (**D**) represent corresponding field of view in a serial section stained with CD68 (green) and CD163 (red).

**Figure 2:**
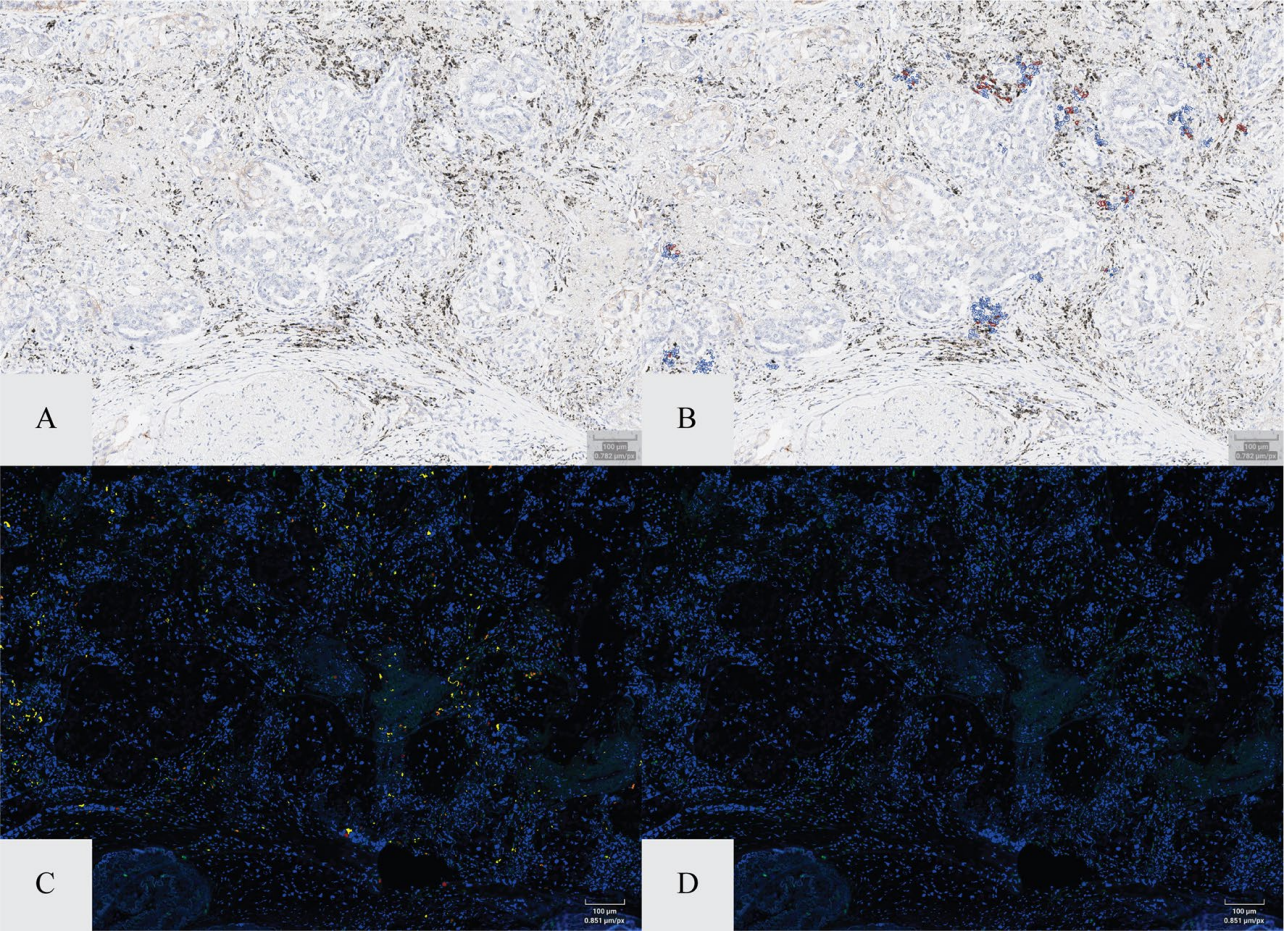
IA vs. double immunofluorescence of lymphocyte identification. Panel (**A**) and (**B**) show PD-L1 stained tumor field of view with and without the lymphocyte labeling (red = PD-L1 positive; blue = PD-L1 negative). Panel (**C**) and (**D**) represent corresponding field of view in a serial section stained with CD3 (green) and CD20 (red).

**Figure 3:**
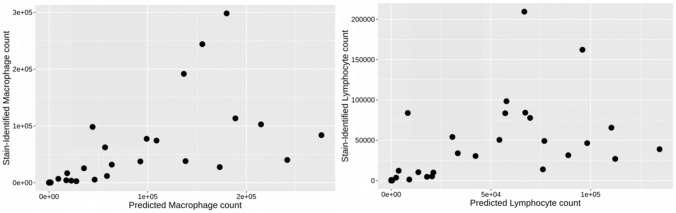
Comparison of immune cell predicted counts vs immunofluorescence counts. Comparison between counts of algorithm-predicted macrophages and lymphocytes vs. counts of staining-identified cells of the same type.

Both the macrophage and lymphocyte IA-based solutions demonstrated acceptable performance in predicting macrophages and lymphocytes in the DAB-conjugated PD-L1 stained sections. Of the 99 NSCLC specimens evaluated, 100% of the tissue cohort exhibited appropriate macrophage and lymphocyte recognition (i.e., ability of algorithm to accurately identify objects as macrophages and lymphocytes only when they correspond to true immune cells in ≥ 90% of the cells evaluated and accurately classify PD-L1 negative immune cells as negative [false positive rate ≤ 10%]) when assessed by the pathologist. Furthermore, 100% of the tissue cohort exhibited appropriate macrophage and lymphocyte identification (i.e., ability of the algorithm to accurately identify macrophages and lymphocytes when present in ≥ 90% of the cells evaluated and accurately classify PD-L1 positive immune cells as positive [false negative rate ≤ 20%]). The IA markups for the macrophage solution predicted macrophages vs. the CD163 and CD68-stain identified macrophages are shown in Fig. 1. The IA markups for the lymphocyte solution predicted lymphocytes and the CD20 and CD3-stain identified lymphocytes are shown in Fig. 2. Figure 3 shows the comparison between the counts of predicted cellular subtypes (macrophages and lymphocytes) and the enumeration of these cells in their IF-stained ‘ground truth’ counterpart section.

The macrophage solution exhibited a significant correlation (r > 0.80, p < 0.001) between the IA macrophage solution predicted macrophage count and the CD163 and CD68-stain identified macrophage counts in the sample cohort (Fig. 1). Moreover, the average percent error between the CD163 and CD68-stain identified macrophage counts and the predicted macrophage count was − 1.28% (Table S3). The lymphocyte solution also exhibited a significant correlation (r > 0.80, p < 0.001) between the IA lymphocyte solution predicted lymphocyte count and the CD20 and CD3-stain identified lymphocyte counts in the sample cohort (Fig. 2). The average percent error between the CD20 and CD3-stain identified lymphocyte counts and the predicted lymphocyte count was − 0.67% (Table S4).

Lastly, the macrophage and lymphocyte solutions proved to be precise by exhibiting an ICC and confidence interval (CI) values ≥ 0.6 for the predicted macrophage count and the predicted lymphocyte count in the three serial sections of the same samples stained for PD-L1 in Part 1 of the study.

#### Digital and manual pathology TPS comparisons

Comparison of the TPS scores from the digital assay with and without applying the combined macrophage and lymphocyte solutions demonstrated that there was no difference in the performance of the algorithm in identifying PD-L1 positive cells. When compared to the manual pathology TPS assessment, the digital TPS scores from the digital assay were substantially equivalent. The concordance in non-treatment (TPS < 1%) and treatment (TPS ≥ 1%) binning according to the digital versus manual pathology TPS evaluations demonstrated that none of the cases changed category with the two different scoring modalities.

## Discussion

There were two main goals of this study. The first was to validate the TI 22C3 NSCLC DA assay performs in accordance to predetermined CLIA criteria. Additionally, to establish the assay is equivalent to manual pathologist scoring, requiring less labor, and bypassing intra- and inter-pathologist variability through standardized and reproducible results with greater precision across repeated scoring. The Tissue Insight 22C3 PDL1 NSCLC DA fulfilled these criteria and was clinically validated (i.e. according to CLIA standards of sensitivity, specificity, accuracy and precision) supporting its adoption as a predictor of checkpoint inhibitor therapy and future use in clinical trials.

Generalized adoption of this digital image analysis solution will allow for better standardization of PD-L1 scoring across different laboratories and allow more accurate outcome studies including cumbersome large sample cohorts. With complexities related to IVD staining performances, various scoring methods (TPS/ tumor cell population versus immune cells/total tumor area versus combined positive scoring [CPS]), and intratumor heterogeneity, many studies have shown substantial differences between pathologists’ interpretation of PD-L1 staining and resulting TPS^2,5,6^. This is not only a challenge related to NSCLC, but a difficulty amongst a multitude of cancer therapies^4,12^. Future refinement may lead to further improvement in prediction algorithms (based on TPS score in NSCLC and CPS in others cancer therapies^12^) for response to checkpoint inhibitor therapy. This approach is based on generating a digital version of a previously developed solution using pathologist interpretation of PD-L1 stained glass slides and, therefore, carries negligible risk to significantly deviate from the current gold standard of checkpoint inhibitor therapy effectiveness prediction. In addition, the elimination of the inter and intra pathologist scoring variability (given the consistency of computer assisted Digital scoring) will greatly improve the reliability of treatment decisions^3–6^.

The second component of this study diverged from the current 22C3 NSCLC scoring paradigms as it added information on the number of immune cells (tissue and alveolar macrophages) in the tumor and stroma compartment as well as their PD-L1 status. As summarized in the introduction section, this could lead to a significant improvement of the current PD-L1 22C3 NSCLC assay yielding a better predictive value for checkpoint inhibitor therapy response^2,8,10^. While this modification of the assay would be a substantial departure from the current scoring algorithm, we believe that the risk associated with its adoption is still extremely low. This is because Tissue Insight’s 22C3 NSCLC is based upon digital reading of sections that are stained according to the conventional IVD PD-L1 22C3 protocol and can thus be scored with either a digital or manual pathology assessment (in order to increase the confidence of adopting laboratories in the digital solution). The supplementary information on macrophages and lymphocytes does not require any modification of the IVD protocol as they are simply based on the addition of macrophage recognition algorithms (one for tissue macrophages and one for alveolar macrophages) to the computational analysis of the images.

In practice, the pathologist using Tissue Insight 22C3 NSCLC, would have access to several layers of information regarding the sections to be assessed for PD-L1 staining. First and foremost, a digitized version of the physical slide would be available for manual scoring, if so desired. Additionally, the pathologist would have the digital scoring of the slide with associated mark-up of positive and negative cells to compare with the manual score and thus ultimately accept or reject the assessment. It is important to emphasize here that, even if the pathologist were to reject the digital scoring, this scoring could aid in manual scoring by providing an accurate cell count. The number of true positive cells could also be adjusted by the pathologist by determining the percentage of false positive and/or false negative determined by the algorithm. The third level of information would consist of the number of PD-L1 positive and negative macrophages and lymphocytes within the tumor and stroma compartments. Although the number and PD-L1 status of immune cells is not part of the calculation of the TPS, identifying macrophages and lymphocytes within the tumor may yield a more accurate determination of the denominator (by counting only tumor cells and not intratumoral immune cells). Furthermore, the number of PD-L1 positive immune cells might help improve the positive predictive value of the current tumor cells PD-L1 staining assessment. Tissue Insight is not intended to replace the pathologist assessment but to be a tool to ease and integrate such an assessment.

In this study, we demonstrated that our proprietary algorithm Tissue Insight can yield comparable results to pathologist interpretation but reduce the inter- and intra-pathologist variability associated with manual scoring. Furthermore, we have also shown that our digital solution in combination with immune recognition algorithms can provide data on the number of macrophages and lymphocytes within tumor and stroma compartments, as well as their immune cells PD-L1 positivity status. This can all aid pathologists with a more reproducible standardized PD-L1 assessment and additional immune microenvironment data to improve patient outcomes. Future work will expand this approach to additional indications, such as squamous cell carcinoma of the head and neck (SSCHN) and gastric and gastric of adrenal carcinomas, where CPS-based decisions of patient treatment make the assessment of immune cells and their PD-L1 positivity status even more crucial to the accuracy of those assays.

## Materials and methods

### Part 1: clinical validation

#### Tissues and IHC staining

Tissues from 66 NSCLC samples (comprised of 20 internally sourced whole tissue blocks and 46 TMA cores from an internally sourced TMA block) were formalin-fixed, paraffin-embedded (FFPE), sectioned at 4 microns, and stained for PD-L1 (22C3) using the clinically verified class I IHC IVD from Dako (anti-PD-L1 [22C3] pharmDx kit: SK006). These 66 stained samples were used to assess the sensitivity, specificity, and accuracy of the digital PD-L1 assay. To assess for the precision of the digital assay, a subset of 4 samples with a range of PD-L1 staining intensities were chosen to have additional serial sections cut and stained for PD-L1 (22C3) on two subsequent staining days, one day stained within the same laboratory and the other day stained at an outside laboratory. The PD-L1 (22C3) IVD was performed on the Dako Autostainer Link 48 platform (Agilent Technologies, Santa Clara, CA) according to the manufacturer’s instrument specific labeling protocol.

#### Digital image acquisition

Stained tissue slides were scanned on the Leica Aperio AT Turbo brightfield scanner (Leica Biosystems, Wetzlar, Germany) to produce high-resolution whole-slide digital images (in .svs format) at 20X magnification. All digital images passed a quality check for acceptable imaging quality (i.e., in focus, absent/minimal scanning or glass slide artifacts).

#### Algorithm development and image analysis application

The proprietary IA solution consists of an algorithm which attempts to properly identify cells, separate tumor and stroma compartments, and classify the PD-L1 staining of cells. This IA solution was developed using a subset of images from the sample cohort (~ 10%) that captured the full range of representative whole tissue and TMA core sample types and PD-L1 staining intensities. Testing assessment of the algorithm’s ability to accurately detect and identify target cells, separate tumor and stroma compartments, and classify PD-L1 staining in target cells (i.e., tumor cell membranes, immune cells) resulted in the pre-defined PD-L1 (22C3) IVD scoring scheme for TPS.

The development samples underwent whole tissue annotations to ensure that appropriate tissue areas were assessed. An analyst manually annotated regions on each digital slide image for IA inclusion (e.g., tumor parenchyma and tumor microenvironment) and exclusion (e.g., unanalyzable tissue such as necrosis, anthracosis, pigment, folding, dust, crush artifacts, or other tissue-specific artifacts and analyzable non-target tissue such as non-neoplastic tissue). A board-certified anatomic pathologist reviewed the annotations of each image and verified their anatomical accuracy. Each sample was then annotated individually for the tumor/stroma separations. Flagship’s proprietary machine learning (ML) algorithms were used to separate the tumor compartment from the stromal compartment of tissues and remove any immune infiltrate from the tumor compartment. This ML technique utilizes all cellular biofeatures measured by the IA solution to determine which cells belong to the tumor and which cells belong to the tissue stroma. An analyst circled small representative areas of tumor and stroma to provide labeled cellular feature data (manual training) to the ML algorithm for model fitting. The tumor/stroma classification algorithm then used this information to classify cells as belonging either to the tumor parenchyma and stromal compartments. All tumor/stroma separations were reviewed by the pathologist and verified as anatomically accurate. The IA solution then defined staining positivity, as staining foci that met a certain staining intensity threshold, which accurately removed background noise and detected true staining. This threshold was set by the analyst and verified by a pathologist for accuracy.

**Figure 4:**
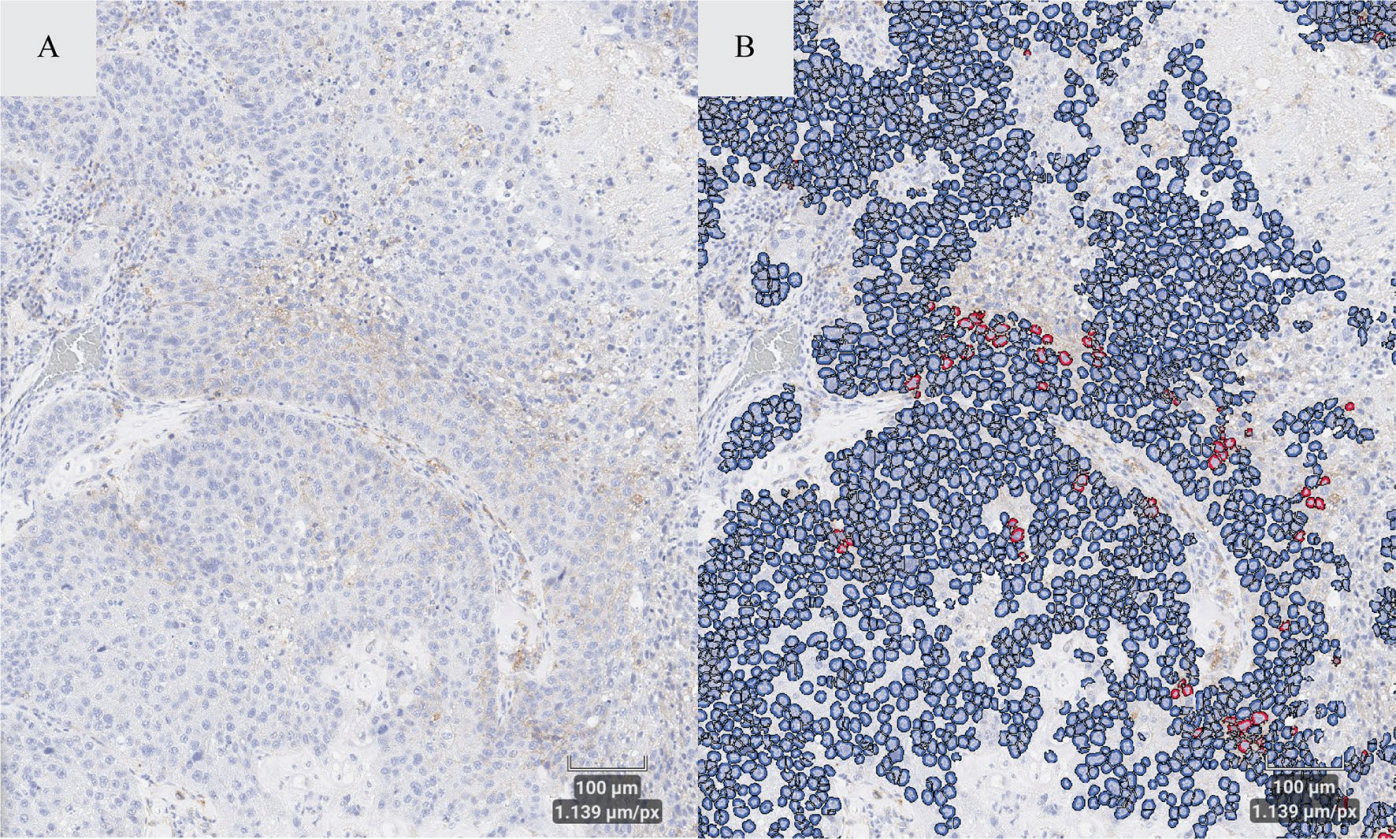
IA algorithm for identifying PD-L1 positive cells in tissue samples. Panel (**A**) shows a field of view of the unmarked PD-L1 stained slide. Panel (**B**) shows the same field of view with labeling of tumor cells in blue (PD-L1 negative tumor cells) and red (PD-L1 positive cells).

**Figure 5:**
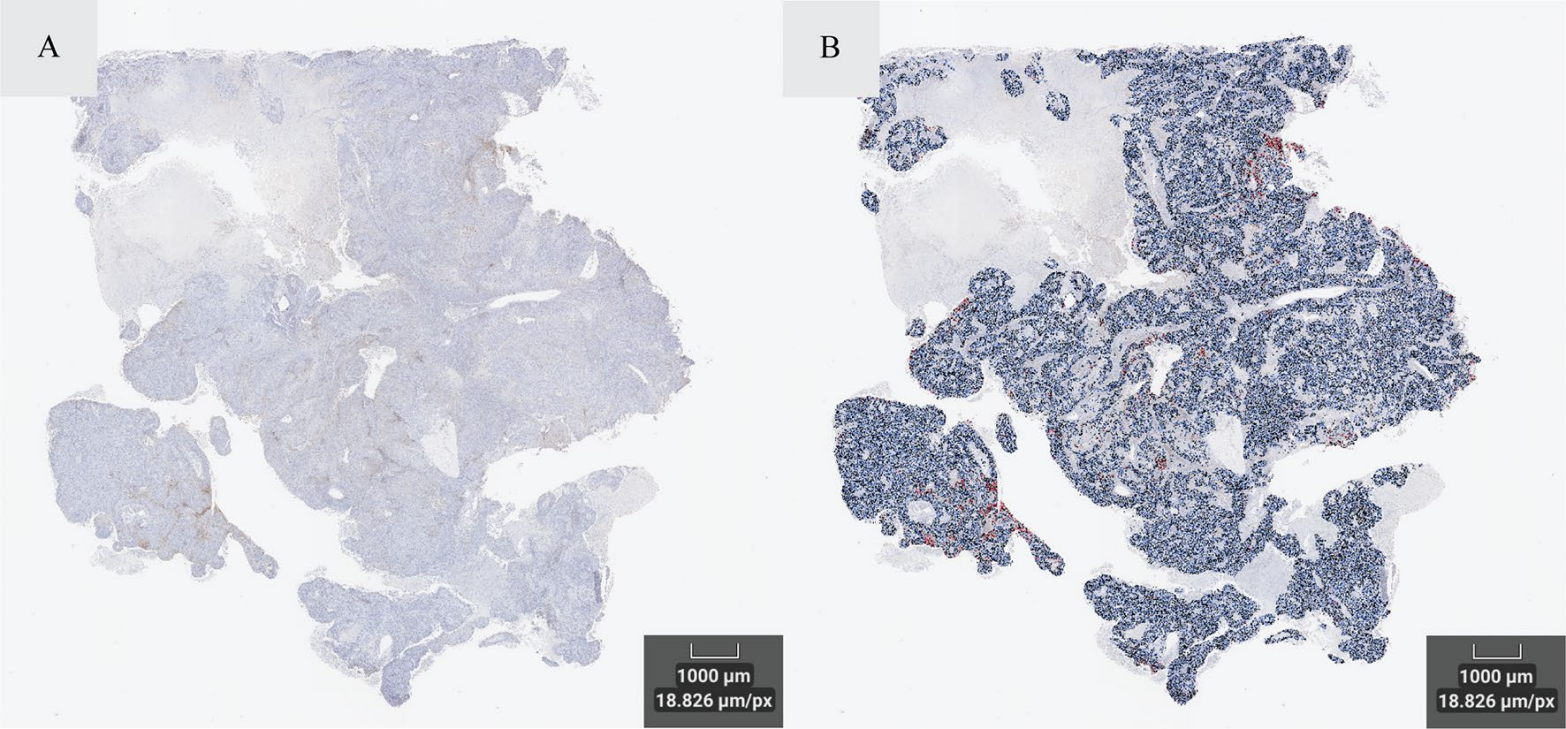
IA algorithm for identifying PD-L1 positive cells in whole tissue samples. Panel (**A**) shows whole tissue image of PD-L1 stained tumor. Panel (**B**) shows the same whole tissue with labeling of tumor cells in blue (PD-L1 negative tumor cells) and red (PD-L1 positive cells).

Development of the IA algorithm involved a fully digital analytical workflow that integrates pathologist review for clinical oversight. Specifically, the pathologist visually reviewed the algorithm performance within a field of view (when using the set threshold) in each of the development samples according to three main criteria: (1) correct identification of cells, (2) correct tumor/stroma separation, and (3) correct identification and classification of PD-L1 (22C3) staining positivity (Fig. 4). The staining threshold was finalized when the algorithm performed appropriately in all development samples. Finalized algorithm settings, including cellular definition, tumor/stroma separation settings, and staining intensity thresholds, were set in the algorithm by an analyst under the oversight of the pathologist. Once the IA solution was finalized, threshold parameters did not change for the remainder of the clinical validation effort. The same finalized IA solution and annotation approach were applied to all the validation samples to assess the performance of the digital assay. In the whole tissue, tumor cell counts were conducted within tumor compartments (Fig. 5).

#### Digital and manual sample scoring

NSCLC samples were evaluated for TPS (%) according to the pre-defined PD-L1 (22C3) IVD scoring scheme: (number of viable tumor cells with 1 + or greater membrane IHC PD-L1 positivity/total number of tumor cells) × 100. TPS values were then binned for PD-L1 expression levels according to the FDA-approved pembrolizumab therapy cut-off values: TPS < 1% (no PD-L1 expression) = no therapy, TPS ≥ 1% (PD-L1 expression) = therapy, TPS ≥ 50% (high PD-L1 expression) = therapy. Each sample was scored for whole tissue TPS using the digital assay and manual pathology assessment on digital images to ensure thorough comparability.

#### Clinical validation acceptance criteria

The sensitivity, specificity, accuracy, and precision of the digital PD-L1 (22C3) assay were assessed in the whole tissue following CLIA guidelines. Ninety percent of the samples were required to pass the specificity and sensitivity criteria. Specificity in each sample was defined as the ability of the algorithm to accurately identify objects as cells only when they corresponded to true cells in ≥ 90% of the cells evaluated and accurately classify PD-L1 negative cells as negative (false positive rate ≤ 10%). Sensitivity in each sample was defined as the ability of the algorithm to accurately identify cells when present in ≥ 90% of the cells evaluated and accurately classify PD-L1 positive cells as positive (false negative rate ≤ 20%). Accuracy required at least 90% of the sample cohort to have concordant digital IA and manual pathology TPS scores that resulted in the same treatment decision point (i.e., treatment [TPS ≥ 1%] versus no treatment [TPS < 1%]). For precision to pass, at least 90% of the sample cohort had to have concordant digital IA and manual pathology TPS scores that resulted in the same treatment decision point across the different staining days within the same laboratory and between different laboratories.

### Part 2: concordance testing

#### Tissues and IHC staining

Ninety-nine different FFPE NSCLC tissue samples (comprised of 25 internally sourced whole tissue blocks and 74 TMA cores from the LC10011b TMA block procured from US Biomax, Inc.) were sectioned at 4 microns and stained for PD-L1 (22C3) using the clinically verified class I IHC IVD from Dako (anti-PD-L1 [22C3] pharmDx kit: SK006). The PD-L1(23C3) IVD was performed on the Dako Autostainer Link 48 platform (Agilent Technologies, Santa Clara, CA) according to the manufacturer’s instrument specific labeling protocol.

#### Digital image acquisition and IA application

Stained tissue slides were scanned on the Leica Aperio AT Turbo brightfield scanner (Leica Biosystems, Wetzlar, Germany) to produce high-resolution whole-slide digital images (in. svs format) at 20X magnification. All digital images passed a quality check for acceptable image quality. The clinically validated digital PD-L (22C3) assay from Part 1 of the study was applied to this sample cohort following the same annotation approach.

#### Digital and manual sample scoring

The NSCLC samples were evaluated on digital images for TPS values by the digital PD-L1 (22C3) assay and manually by three different pathologists using the pre-defined IVD PD-L1 (22C3) scoring scheme. Three repeated scoring (with at least a one-week washout between assessments) were conducted by each pathologist and the digital assay.

#### Statistical analysis

Statistical analyses were performed with the R software (version 3.6.3, R Core Team, 2020). Digital-to-manual PD-L1 (22C3) scoring agreement was assessed for the first scoring by Pearson’s moment correlation of the TPS from the digital assay with the TPS from each pathologist. Inter-pathologist (manual-to-manual) TPS concordance for the first scoring was also assessed using Pearson’s correlation. The Bonferroni correction for multiple comparisons was applied for each concordance assessment. Thus, p < 0.017 (0.05/3) was required for a statistically significant digital-to-manual and manual-to-manual TPS concordance.

Intra-pathologist and intra-digital concordance across the three repeated scoring were assessed by ICC analysis. A linear, random mixed-effects model was fit to the TPS endpoint, and analysis of variance was used to assess the proportion of variance attributable to the assessment day, unique samples, and residual error. The intra-pathologist and intra-digital concordance were considered acceptable for precision benchmarks if an inter-day ICC score (1 – [proportion of variance due to repeated scoring + proportion of residual variance]) was ≥ 0.6. Furthermore, an excess of 1000 simulations of this assessment were performed to generate a 95% CI around this ICC estimate. The concordance should be considered acceptable if the values of this range do not drop below the 0.6 mark (i.e., the lower and upper bound of the 95% CI must be ≥ 0.6). Having an ICC score greater than 0.6 means that > 60% of the variance in the TPS endpoint is attributable to the inter-sample variability and residual error, as opposed to the variance resulting from repeated scoring. Based on the 95% confidence interval of the ICC estimate, values less than 0.5, between 0.5 and 0.75, between 0.75 and 0.9, and greater than 0.9 are indicative of poor, moderate, good, and excellent reliability, respectively.

### Part 3: validation of the macrophage and lymphocyte IA-based solutions

#### Classifier development and model fitting

A training set of data, representing quantified cellular features labeled with a cell’s macrophage or lymphocyte status, was derived separately for the two immune-cell subtype classifiers. To label macrophages, an independent set of NSCLC tissue sections were stained with a CD68/CD163 duplex assay and imaged using a fluorescent scanner. Within these scanned images, the staining was stripped off, and the images reanalyzed with a SP263 PD-L1 assay, and re-scanned. The two resulting images were co-registered using a fine-alignment algorithm that provided direct cell-to-cell registration. Cells expressing staining intensity for either CD68 or CD163 were labeled as macrophages, while all others were labeled ‘non-macrophages’. This status was used to annotate the set of data derived from the cellular feature quantification of the PD-L1 section image. Similarly, training lymphocyte data was derived using the same series of PD-L1 stained tissue sections which were annotated under the supervision of a board-certified MD pathologist.

A series of statistical models were fit to these data and tested against an alternative verification set of samples, for which already had enumeration of the number of macrophages and lymphocytes. The models which performed the most accurately under these circumstances proceeded to be used in the concordance study.

#### Tissues and IHC staining

The same tissue samples used in Part 2 of the study (concordance testing) along with additional whole tissue samples were examined for the evaluation of the macrophage and lymphocyte IA-based solutions. Three additional consecutive serial sections were cut (at 4 microns) from each of the 101 FFPE NSCLC tissue samples (comprised of 9 whole tissue blocks and 74 TMA cores from the concordance testing and 18 different whole tissue samples to replace the 16 whole tissue blocks from the concordance testing that were exhausted). The first serial section was stained for PD-L1 (22C3) using the clinically verified class I IHC IVD from Dako (anti-PD-L1 [22C3] pharmDx kit: SK006). The PD-L1 (22C3) IVD was performed on the Dako Autostainer Link 48 platform (Agilent Technologies, Santa Clara, CA) according to the manufacturer’s instrument specific labeling protocol.

The second serial section was dual IF-stained for CD68 (Cy5 chromogen) using the clinically verified class I IVD from Roche (Ventana CONFIRM anti-CD68 [KP-1] antibody: 790–2931; Ventana Discovery OmniMap anti-Ms HRP kit: 760–4310) and CD163 (FITC chromogen). The CD68 (KP-1) and CD163 (MRQ-26) IF-duplex staining was performed on the Ventana BenchMark ULTRA Autostainer (Roche Diagnostics, Risch-Rotkreuz, Switzerland) according to the manufacturer’s instrument specific labeling protocol: antigen retrieval = pH8 for 64 min at 90 °C, H202 = 4 min, antibody concentration = ready to use predilute incubated for 16 min, DAB chromogen (Ventana: 760–159) = 8 min, hematoxylin counterstain (Ventana: 760–2021) = 8 min, bluing post-counterstain (Ventana: 760–2037) = 8 min.

The third serial section was dual IF-stained for CD20 (Cy5 chromogen) using the clinically verified class I IVD from Roche (Ventana anti-CD20 [KP-1] antibody: 790–2931; Ventana Discovery OmniMap anti-Ms HRP kit: 760–4310) and CD3 (FITC chromogen) using the CD3 2VG6; 790–4341 antibody; Ventana. The CD20 (CLONE) and CD3 (CLONE) IF-duplex staining was performed on the [Ventana BenchMark ULTRA Autostainer (Roche Diagnostics, Risch-Rotkreuz, Switzerland) according to the manufacturer’s instrument specific labeling protocol: antigen retrieval = pH 8 for 64 min at 90 °C, H202 = 4 min, antibody concentration = ready to use predilute incubated for 16 min, DAB chromogen (Ventana: 760–159) = 8 min, hematoxylin counterstain (Ventana: 760–2021) = 8 min, bluing post-counterstain (Ventana: 760–2037) = 8 min.

#### Digital image acquisition and IA application

The PD-L1-stained tissue slides were scanned on the Leica Aperio AT Turbo brightfield scanner (Leica Biosystems, Wetzlar, Germany) and the CD68/CD163 and CD20/CD3 duplex IF-stained tissue slides were scanned on the Vectra Polaris scanner (Akoya Biosciences, Marlboro, MA]) to produce high-resolution whole-slide digital images (in .svs format) at 20 × magnification. All digital images passed a quality check for acceptable image quality.

**Figure 6:**
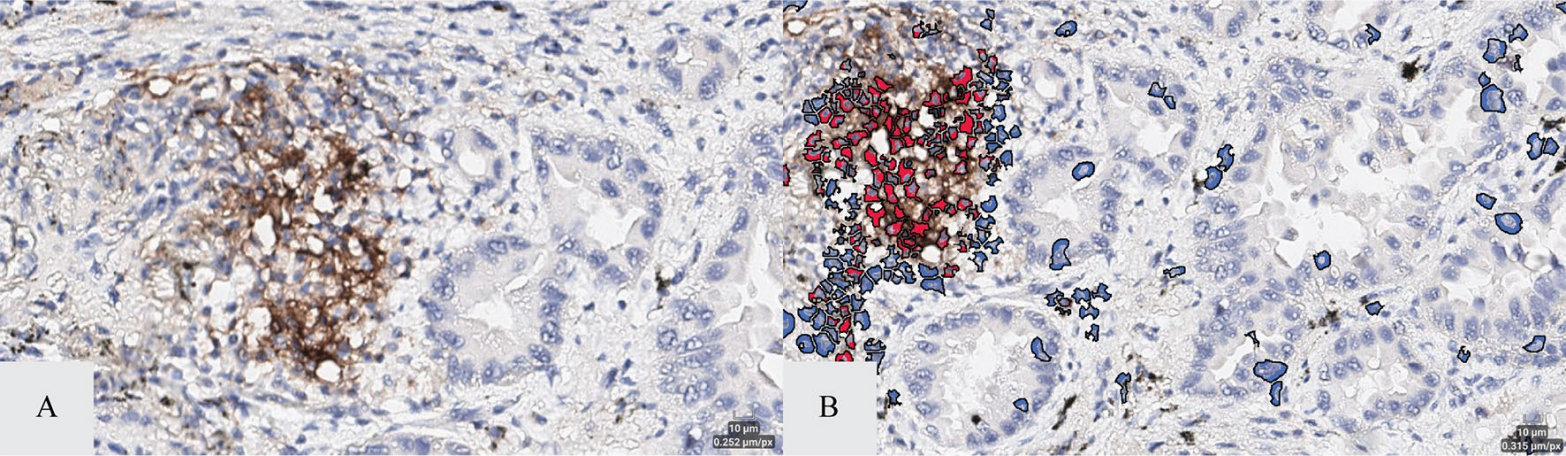
Macrophage identification and PD-L1 positivity IA solution. Panel (**A**) shows a field of view of intra-tumoral macrophages. Panel (**B**) shows the same field of view with labeled macrophages (as identified by the recognition algorithm) with and without PD-L1 staining (red = PD-L1 positive; blue = PD-L1 negative).

The clinically validated digital PD-L1 (22C3) assay from Part 1 of the study was applied to PD-L1 (22C3)-stained sections in the whole tissue following the same annotation approach. To account for the contribution of macrophages in the PD-L1 (22C3) TPS assessment, we applied a previously developed macrophage specific IA solution to the clinically validated digital PD-L1 (22C3) assay. This macrophage solution allows for the detection of both tissue and alveolar macrophages in the NSCLC tissues which were quantified in the tumor compartment of each PD-L1 (22C3) stained section (Fig. 6). The number of CD68-positive stained macrophages and the number of CD163-positive stained macrophages in the CD68/CD163 duplex IF-stained sections were also quantified using our proprietary IA software and served as the ground truth against which we compared the macrophage solution as applied to the clinically validated digital PD-L1 (22C3) assay.

**Figure 7:**
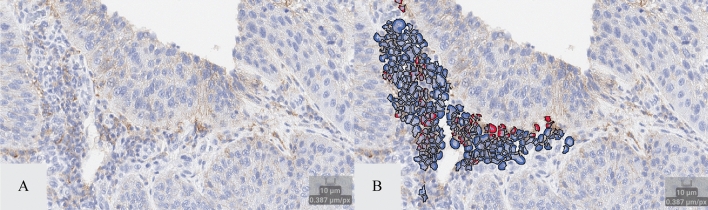
Lymphocyte identification and PD-L1 positivity IA solution. Panel (**A**) shows a field of view of stromal lymphocytes. Panel (**B**) shows the same field of view with labeled lymphocytes (as identified by the recognition algorithm) with and without PD-L1 staining (red = PD-L1 positive; blue = PD-L1 negative).

To account for the contribution of lymphocytes in the PD-L1 (22C3) TPS assessment, we applied a previously developed lymphocyte specific IA solution to the clinically validated digital PD-L1 (22C3) assay. This lymphocyte solution allows for the detection of lymphocytes in the NSCLC tissues which were quantified in the tumor compartment of each PD-L1 (22C3)-stained section (Fig. 7). The number of CD20-positive stained lymphocytes and the number of CD3-positive stained lymphocytes in the CD20/CD3 duplex IF-stained sections were also quantified using our proprietary IA software and served as the ground truth against which we compared the lymphocyte solution as applied to the clinically validated digital PD-L1 (22C3) assay.

#### Digital and manual sample scoring

The PD-L1 (22C3) sections were manually evaluated for TPS values in the tumor compartment by an anatomic pathologist using the predefined PD-L1 (22C3) IVD scoring scheme. The TPS values were also calculated in the tumor compartment using the digital PD-L1 (22C3) assay with and without applying the combined macrophage and lymphocyte IA-based solutions.

#### Algorithm performance assessment

The sensitivity, specificity, accuracy, and precision of the macrophage and lymphocyte IA-based solutions were assessed within the tumor compartments of each sample. Ninety percent of the samples were required to pass the specificity and sensitivity criteria. Specificity in each sample was defined as the ability of the algorithm to accurately identify objects as macrophages and lymphocytes only when they corresponded to true immune cells in ≥ 90% of the cells evaluated and accurately classify PD-L1 negative immune cells as negative (false positive rate ≤ 10%). Sensitivity in each sample was defined as the ability of the algorithm to accurately identify macrophages and lymphocytes when present in ≥ 90% of the cells evaluated and accurately classify PD-L1 positive immune cells as positive (false negative rate ≤ 20%).

For the macrophage solution, accuracy required a significant correlation (Pearson’s r ≥ 0.80) between the number of tissue and alveolar macrophages predicted by the macrophage solution in the PD-L1-stained images with the number of CD68- and CD163-positive macrophages in the CD68/CD163 dual IF-stained images. For the lymphocyte solution, accuracy required a significant correlation (Pearson’s r ≥ 0.80) between the number of lymphocytes predicted by the lymphocyte solution in the PD-L1-stained images with the number of CD20- and CD3-positive lymphocytes in the CD20/CD3 dual IF-stained images.

For the precision assessment, three serial sections of the same samples stained for PD-L1 in Part 1 of the study were assessed for the number of total macrophages (tissue and alveolar) as detected by the IA-based macrophage solution and the number of total lymphocytes as detected by the IA-based lymphocyte solution. The ICC of the number of macrophages predicted across the three precision days and the ICC for the number of lymphocytes predicted across the three precision days were calculated to describe the similarity of related measurements for each respective IA-based solution. The macrophage and lymphocyte solutions were considered acceptable for precision if their respective ICC score (1 – [proportion of variance due to day + proportion of residual variance]) was greater than 0.6 and if the CI values did not drop below the 0.6 mark (i.e., lower and upper bound of the 95% CI must be ≥ 0.6).

#### Statistical analysis

Pearson’s correlation analyses were conducted to assess for accuracy of the macrophage and lymphocyte IA-based solutions and ICC analyses were conducted for the precision assessment of each IA-based solution. Comparison of the digital TPS scores from the digital assay with and without applying the combined macrophage and lymphocyte solutions was also assessed. Lastly, we conducted a follow-up digital-to-manual concordance testing on the PD-L1-stained images using Pearson’s correlation analyses of the manual pathology TPS values versus the digital TPS values calculated from the digital PD-L1 (22C3) assay with and without applying the combined macrophage and lymphocyte solutions. Concordance in non-treatment (TPS < 1%) and treatment (TPS ≥ 1%) binning according to the digital versus manual pathology TPS evaluations were also evaluated.

## Supplementary Information

Supplementary Tables.

## Data Availability

The datasets used and/or analyzed during the current study are available from the corresponding author upon reasonable request.
