# Supplementary material for: A digital assay for programmed death-ligand 1 (22C3) quantification combined with immune cell recognition algorithms in non-small cell lung cancer
Source: Sci Rep. 2022 Jun 13;12:9745. doi: 10.1038/s41598-022-12697-1 (PMC9192755; doi:10.1038/s41598-022-12697-1)
Supplement: Supplementary file 1 — Supplementary Tables. [file 41598_2022_12697_MOESM1_ESM.docx]

**Supplemental Tables**

**Table S1.** Manual to Manual concordance by three pathologists.

| Sample Name | Slide Name | Digital Percent positive cells | Manual Read 1 | Manual Read 2 | Manual Read 3 |
| --- | --- | --- | --- | --- | --- |
| NSCLC 48 | 1051886 | 3.649 | 0 | 10 | 10 |
| NSCLC 58 | 1051885 | 1.916 | 0 | 5 | <1 |
| NSCLC 65 | 1051884 | 53.846 | NA | 40 | 50 |
| NSCLC 90 | 1051883 | 0.298 | 0 | <1 | <1 |
| NSCLC 93 | 1051882 | 0.184 | 0 | 0 | 0 |
| NSCLC 93-1 | 1051881 | 0.21 | 0 | <1 | <1 |
| NSCLC 95-2 | 1051880 | 5.362 | 20 | 50 | 80 |
| NSCLC 98 | 1051879 | 1.62 | 0 | 10 | 3 |
| NSCLC 98-2 | 1051878 | 15.451 | 0 | 50 | 20 |
| NSCLC 104 | 1051877 | 3.545 | 1 | 1 | 5 |
| NSCLC 116 | 1051876 | 79.496 | 70 | 95 | 90 |
| NSCLC 117 | 1051875 | 1.962 | 0 | <1 | 5 |
| NSCLC CA 142 | 1051874 | 85.44 | 75 | 100 | 90 |
| NSCLC CA 143 | 1051873 | 0.0964 | 0 | 0 | 0 |
| Lung SCC 2 | 1051866 | 56.102 | 40 | 95 | 80 |
| Lung SCC 3 | 1051865 | 81.018 | 75 | 95 | 80 |
| Lung SCC 4 | 1051864 | 0.922 | 0 | <1 | <1 |
| Lung SCC 8 | 1051947 | 85.446 | 90 | 100 | 100 |
| Lung SCC 9 | 1051863 | 88.618 | 90 | 95 | 100 |
| Lung Adeno 6 | 1051872 | 15.852 | 4 | 60 | 70 |
| Lung Adeno 11 | 1051869 | 21.89 | 7 | 50 | 60 |
| Lung Adeno 12 | 1051871 | 19.828 | 0 | 10 | 2 |
| Lung Adeno 19 | 1051870 | 3.943 | 1 | 5 | 8 |
| Lung Adeno 27 | 1051868 | 3.738 | 0 | 10 | 5 |
| Lung Adeno 29 | 1051867 | 27.51 | 25 | 75 | 80 |
| LC10011b - A1 | 1051862_WT_0101 | 9.181 | 0 | <1 | 0 |
| LC10011b - A2 | 1051862_WT_0102 | 6.13 | 0 | 1 | 5 |
| LC10011b - A3 | 1051862_WT_0103 | 1.589 | 0 | <1 | 0 |
| LC10011b - A4 | 1051862_WT_0104 | 27.5 | 0 | 0 | 0 |
| LC10011b - A5 | 1051862_WT_0105 | 90.273 | 85 | 100 | 100 |
| LC10011b - A6 | 1051862_WT_0108 | 0.567 | 0 | 0 | 0 |
| LC10011b - A7 | 1051862_WT_0109 | 3.44 | 0 | 1 | <1 |
| LC10011b - A8 | Missing Core | Missing Core | NA | 0 | 0 |
| LC10011b - A9 | 1051862_WT_0111 | 2.823 | 0 | 0 | 0 |
| LC10011b - A10 | 1051862_WT_0112 | 18.867 | 0 | 20 | 20 |
| LC10011b - B1 | 1051862_WT_0201 | 59.773 | 30 | 50 | 60 |
| LC10011b - B2 | 1051862_WT_0202 | 35.167 | 35 | 75 | 80 |
| LC10011b - B3 | 1051862_WT_0203 | 37.659 | 5 | 25 | 40 |
| LC10011b - B4 | 1051862_WT_0204 | 1.183 | 0 | <1 | 0 |
| LC10011b - B5 | 1051862_WT_0205 | 63.638 | 50 | 90 | 80 |
| LC10011b - B6 | 1051862_WT_0208 | 55.414 | 30 | 50 | 60 |
| LC10011b - B7 | 1051862_WT_0209 | 23.121 | 8 | 30 | 35 |
| LC10011b - B8 | 1051862_WT_0210 | 50.557 | 25 | 50 | 60 |
| LC10011b - B9 | 1051862_WT_0211 | 4.39 | 0 | 1 | 1 |
| LC10011b - B10 | 1051862_WT_0212 | 36.005 | 30 | 90 | 90 |
| LC10011b - C1 | 1051862_WT_0301 | 27.217 | 10 | 75 | 80 |
| LC10011b - C2 | 1051862_WT_0302 | 4 | 0 | 0 | 0 |
| LC10011b - C3 | 1051862_WT_0303 | 0.104 | 0 | <1 | 0 |
| LC10011b - C4 | 1051862_WT_0304 | 26.186 | 35 | 80 | 70 |
| LC10011b - C5 | 1051862_WT_0305 | 2.52 | 0 | 5 | 5 |
| LC10011b - C6 | 1051862_WT_0308 | 24.682 | 12 | 90 | 100 |
| LC10011b - C7 | 1051862_WT_0309 | 2.96 | 0 | 10 | 2 |
| LC10011b - C8 | 1051862_WT_0310 | 0.0882 | 0 | 0 | 0 |
| LC10011b - C9 | 1051862_WT_0311 | 55.654 | 30 | 80 | 70 |
| LC10011b - C10 | 1051862_WT_0312 | 2.117 | 0 | 1 | <1 |
| LC10011b - D1 | 1051862_WT_0401 | 3.179 | 0 | 5 | 10 |
| LC10011b - D2 | 1051862_WT_0402 | 90.289 | 60 | 95 | 90 |
| LC10011b - D3 | 1051862_WT_0403 | 75.194 | 35 | 100 | 95 |
| LC10011b - D4 | 1051862_WT_0404 | 6.683 | 0 | 20 | 10 |
| LC10011b - D5 | 1051862_WT_0405 | 0.132 | 0 | 1 | <1 |
| LC10011b - D6 | 1051862_WT_0408 | 9.819 | 3 | 40 | 30 |
| LC10011b - D7 | 1051862_WT_0409 | 85.753 | 70 | 95 | 70 |
| LC10011b - D8 | 1051862_WT_0410 | 89.758 | 30 | 95 | 90 |
| LC10011b - D9 | 1051862_WT_0411 | 36.359 | 0 | 30 | 20 |
| LC10011b - D10 | 1051862_WT_0412 | 0.769 | 0 | 1 | 0 |
| LC10011b - E1 | 1051862_WT_0501 | 13.547 | 0 | 50 | 60 |
| LC10011b - E2 | 1051862_WT_0502 | 4.643 | 5 | 10 | 10 |
| LC10011b - E3 | 1051862_WT_0503 | 2.414 | 0 | 5 | 5 |
| LC10011b - E4 | 1051862_WT_0504 | 88.184 | 90 | 100 | 100 |
| LC10011b - E5 | 1051862_WT_0505 | 10.64 | 0 | 0 | <1 |
| LC10011b - E6 | 1051862_WT_0508 | 7.308 | 0 | 5 | 2 |
| LC10011b - E7 | 1051862_WT_0509 | 4.15 | 5 | 5 | 5 |
| LC10011b - E8 | 1051862_WT_0510 | 2.029 | 0 | 5 | 10 |
| LC10011b - E9 | 1051862_WT_0511 | 67.403 | 85 | 100 | 100 |
| LC10011b - E10 | 1051862_WT_0512 | 15.377 | 0 | 0 | 0 |
| LC10011b - F1 | 1051862_WT_0601 | 46.997 | 50 | 75 | 80 |
| LC10011b - F2 | 1051862_WT_0602 | 87.37 | 80 | 100 | 90 |
| LC10011b - F3 | 1051862_WT_0603 | 29.496 | 0 | 10 | 10 |
| LC10011b - F4 | 1051862_WT_0604 | 3.432 | 0 | 1 | 0 |
| LC10011b - F5 | 1051862_WT_0605 | 2.199 | 0 | 5 | <1 |
| LC10011b - F6 | 1051862_WT_0608 | 29.581 | 15 | 50 | 70 |
| LC10011b - F7 | 1051862_WT_0609 | 87.865 | 95 | 100 | 100 |
| LC10011b - F8 | 1051862_WT_0610 | 19.525 | 0 | 10 | 5 |
| LC10011b - F9 | 1051862_WT_0611 | 2.072 | 0 | 1 | 0 |
| LC10011b - F10 | 1051862_WT_0612 | 4.06 | 3 | 0 | 0 |
| LC10011b - G1 | 1051862_WT_0701 | 3.638 | 0 | 25 | 15 |
| LC10011b - G2 | 1051862_WT_0702 | 9.677 | NA | 1 | 1 |
| LC10011b - G3 | 1051862_WT_0703 | 27.992 | 0 | 0 | 0 |
| LC10011b - G4 | 1051862_WT_0704 | 3.475 | 0 | 10 | 5 |
| LC10011b - G5 | 1051862_WT_0705 | 5.066 | NA | 0 | 0 |
| LC10011b - G6 | 1051862_WT_0708 | 2.498 | 0 | 5 | 2 |
| LC10011b - G7 | 1051862_WT_0709 | 28 | NA | 0 | 0 |
| LC10011b - G8 | 1051862_WT_0710 | 4.211 | NA | 0 | 0 |
| LC10011b - G9 | 1051862_WT_0711 | 1.208 | 0 | <1 | <1 |
| LC10011b - G10 | 1051862_WT_0712 | 44.402 | 4 | 30 | 40 |
| LC10011b - H1 | 1051862_WT_0801 | 29.296 | 0 | 30 | 40 |
| LC10011b - H2 | 1051862_WT_0802 | 0.273 | 0 | 0 | <1 |
| LC10011b - H3 | 1051862_WT_0803 | 1.893 | 0 | 1 | <1 |
| LC10011b - H4 | 1051862_WT_0804 | 2.804 | 0 | 0 | 0 |
| LC10011b - H5 | 1051862_WT_0805 | 16.37 | 0 | 5 | 2 |
| LC10011b - H6 | 1051862_WT_0808 | 3.863 | 0 | 10 | 5 |
| LC10011b - H7 | 1051862_WT_0809 | 0.219 | 0 | 0 | 0 |
| LC10011b - H8 | 1051862_WT_0810 | 0.256 | 0 | 0 | 0 |
| LC10011b - H9 | 1051862_WT_0811 | 10.842 | 0 | 1 | <1 |
| LC10011b - H10 | 1051862_WT_0812 | 21.81 | 0 | 0 | 0 |

**Table S2.** Intra-digital TPS concordance.

| Sample Name | Slide Name | Digital Percent positive cells | Manual Read 1 | Manual Read 2 | Manual Read 3 | Digital TPS minus Macrophage 1 | Digital TPS minus Macrophage 2 | Digital TPS minus Macrophage 3 | Digital TPS minus Macrophage 4 | Alveolar Macrophages Removed |
| --- | --- | --- | --- | --- | --- | --- | --- | --- | --- | --- |
| Lung SCC 9 | 1051863 | 88.618 | 90 | 95 | 100 | 88.774 | 90.301 | 88.618 | 88.618 | 86.94 |
| Lung SCC 4 | 1051864 | 0.922 | 0 | <1 | <1 | 1.16 | 1.295 | 0.922 | 0.922 | 0.578 |
| Lung SCC 3 | 1051865 | 81.018 | 75 | 95 | 80 | 81.075 | 80.035 | 81.023 | 81.015 | 63.273 |
| Lung SCC 2 | 1051866 | 56.102 | 40 | 95 | 80 | 58.215 | 62.828 | 56.111 | 56.072 | 52.102 |
| Lung Adeno 29 | 1051867 | 27.51 | 25 | 75 | 80 | 32.397 | 35.058 | 27.523 | 27.513 | 13.065 |
| Lung Adeno 27 | 1051868 | 3.738 | 0 | 10 | 5 | 4.639 | 4.527 | 3.745 | 3.74 | 2.032 |
| Lung Adeno 11 | 1051869 | 21.89 | 7 | 50 | 60 | 22.155 | 23.362 | 21.891 | 21.891 | 19.13 |
| Lung Adeno 19 | 1051870 | 3.943 | 1 | 5 | 8 | 5.46 | 4.668 | 3.944 | 3.946 | 1.967 |
| Lung Adeno 12 | 1051871 | 19.828 | 0 | 10 | 2 | 27.811 | 30.268 | 19.936 | 19.861 | 16.231 |
| Lung Adeno 6 | 1051872 | 15.852 | 4 | 60 | 70 | 20.225 | 22.208 | 15.879 | 15.867 | 7.757 |
| NSCLC CA 143 | 1051873 | 0.0964 | 0 | 0 | 0 | 0.0971 | 0.0966 | 0.0964 | 0.0965 | 0.0962 |
| NSCLC CA 142 | 1051874 | 85.44 | 75 | 100 | 90 | 85.851 | 85.995 | 85.44 | 85.44 | 87.301 |
| NSCLC 117 | 1051875 | 1.962 | 0 | <1 | 5 | 1.934 | 2.009 | 1.973 | 1.961 | 1.644 |
| NSCLC 116 | 1051876 | 79.496 | 70 | 95 | 90 | 80.755 | 82.834 | 79.499 | 79.498 | 71.262 |
| NSCLC 104 | 1051877 | 3.545 | 1 | 1 | 5 | 3.814 | 3.926 | 3.546 | 3.546 | 3.029 |
| NSCLC 98-2 | 1051878 | 15.451 | 0 | 50 | 20 | 20.565 | 31.208 | 15.463 | 15.485 | 13.386 |
| NSCLC 98 | 1051879 | 1.62 | 0 | 10 | 3 | 1.706 | 1.816 | 1.621 | 1.622 | 1.21 |
| NSCLC 95-2 | 1051880 | 5.362 | 20 | 50 | 80 | 6.652 | 7.3 | 5.361 | 5.364 | 2.029 |
| NSCLC 93-1 | 1051881 | 0.21 | 0 | <1 | <1 | 0.216 | 0.219 | 0.21 | 0.211 | 0.177 |
| NSCLC 93 | 1051882 | 0.184 | 0 | 0 | 0 | 0.23 | 0.254 | 0.185 | 0.185 | 0.142 |
| NSCLC 90 | 1051883 | 0.298 | 0 | <1 | <1 | 0.364 | 0.408 | 0.298 | 0.299 | 0.0806 |
| NSCLC 65 | 1051884 | 53.846 | NA | 40 | 50 | 53.846 | 53.846 | 53.846 | 53.846 | 39.286 |
| NSCLC 58 | 1051885 | 1.916 | 0 | 5 | <1 | 2.024 | 2.212 | 1.916 | 1.916 | 1.689 |
| NSCLC 48 | 1051886 | 3.649 | 0 | 10 | 10 | 4.227 | 4.837 | 3.656 | 3.666 | 1.629 |
| Lung SCC 8 | 1051947 | 85.446 | 90 | 100 | 100 | 86.638 | 89.832 | 85.452 | 85.449 | 75.396 |
| LC10011b - A1 | 1051862_WT_0101 | 9.181 | 0 | <1 | 0 | 9.693 | 8.163 | 9.181 | 9.181 | 9.052 |
| LC10011b - A2 | 1051862_WT_0102 | 6.13 | 0 | 1 | 5 | 6.351 | 6.747 | 6.086 | 6.13 | 2.811 |
| LC10011b - A3 | 1051862_WT_0103 | 1.589 | 0 | <1 | 0 | 1.524 | 1.147 | 1.589 | 1.589 | 1.589 |
| LC10011b - A4 | 1051862_WT_0104 | 27.5 | 0 | 0 | 0 | 27.993 | 30.077 | 27.5 | 27.5 | 27.5 |
| LC10011b - A5 | 1051862_WT_0105 | 90.273 | 85 | 100 | 100 | 90.287 | 90.897 | 90.273 | 90.273 | 78.248 |
| LC10011b - A6 | 1051862_WT_0108 | 0.567 | 0 | 0 | 0 | 0.509 | 0.51 | 0.567 | 0.567 | 0.567 |
| LC10011b - A7 | 1051862_WT_0109 | 3.44 | 0 | 1 | <1 | 3.515 | 3.405 | 3.44 | 3.44 | 2.826 |
| LC10011b - A9 | 1051862_WT_0111 | 2.823 | 0 | 0 | 0 | 2.717 | 3.017 | 2.823 | 2.823 | 2.823 |
| LC10011b - A10 | 1051862_WT_0112 | 18.867 | 0 | 20 | 20 | 18.929 | 19.035 | 18.84 | 18.867 | 18.871 |
| LC10011b - B1 | 1051862_WT_0201 | 59.773 | 30 | 50 | 60 | 60.142 | 59.307 | 59.85 | 59.773 | 59.816 |
| LC10011b - B2 | 1051862_WT_0202 | 35.167 | 35 | 75 | 80 | 35.638 | 31.751 | 35.167 | 35.167 | 33.46 |
| LC10011b - B3 | 1051862_WT_0203 | 37.659 | 5 | 25 | 40 | 37.607 | 37.822 | 37.655 | 37.659 | 36.976 |
| LC10011b - B4 | 1051862_WT_0204 | 1.183 | 0 | <1 | 0 | 1.239 | 1.123 | 1.184 | 1.183 | 1.183 |
| LC10011b - B5 | 1051862_WT_0205 | 63.638 | 50 | 90 | 80 | 64.457 | 65.796 | 63.631 | 63.638 | 43.71 |
| LC10011b - B6 | 1051862_WT_0208 | 55.414 | 30 | 50 | 60 | 55.041 | 51.446 | 55.395 | 55.414 | 54.395 |
| LC10011b - B7 | 1051862_WT_0209 | 23.121 | 8 | 30 | 35 | 23.184 | 20.982 | 23.128 | 23.121 | 22.949 |
| LC10011b - B8 | 1051862_WT_0210 | 50.557 | 25 | 50 | 60 | 50.481 | 45.643 | 50.572 | 50.557 | 44.691 |
| LC10011b - B9 | 1051862_WT_0211 | 4.39 | 0 | 1 | 1 | 4.498 | 4.56 | 4.394 | 4.39 | 4.391 |
| LC10011b - B10 | 1051862_WT_0212 | 36.005 | 30 | 90 | 90 | 35.482 | 33.535 | 36.005 | 36.005 | 26.982 |
| LC10011b - C1 | 1051862_WT_0301 | 27.217 | 10 | 75 | 80 | 27.307 | 26.606 | 27.196 | 27.217 | 26.509 |
| LC10011b - C2 | 1051862_WT_0302 | 4 | 0 | 0 | 0 | 3.973 | 4.249 | 4 | 4 | 4 |
| LC10011b - C3 | 1051862_WT_0303 | 0.104 | 0 | <1 | 0 | 0.113 | 0.151 | 0.104 | 0.104 | 0.104 |
| LC10011b - C4 | 1051862_WT_0304 | 26.186 | 35 | 80 | 70 | 26.213 | 28.837 | 26.217 | 26.186 | 19.991 |
| LC10011b - C5 | 1051862_WT_0305 | 2.52 | 0 | 5 | 5 | 2.358 | 2.459 | 2.52 | 2.52 | 2.394 |
| LC10011b - C6 | 1051862_WT_0308 | 24.682 | 12 | 90 | 100 | 25.386 | 24.625 | 24.706 | 24.682 | 23.787 |
| LC10011b - C7 | 1051862_WT_0309 | 2.96 | 0 | 10 | 2 | 2.84 | 2.87 | 2.96 | 2.96 | 2.366 |
| LC10011b - C8 | 1051862_WT_0310 | 0.0882 | 0 | 0 | 0 | 0 | 0 | 0.0882 | 0.0882 | 0.0882 |
| LC10011b - C9 | 1051862_WT_0311 | 55.654 | 30 | 80 | 70 | 57.324 | 59.946 | 55.673 | 55.654 | 42.196 |
| LC10011b - C10 | 1051862_WT_0312 | 2.117 | 0 | 1 | <1 | 2.384 | 2.162 | 2.124 | 2.117 | 2.045 |
| LC10011b - D1 | 1051862_WT_0401 | 3.179 | 0 | 5 | 10 | 3.287 | 2.824 | 3.109 | 3.179 | 3.179 |
| LC10011b - D2 | 1051862_WT_0402 | 90.289 | 60 | 95 | 90 | 90.37 | 91.862 | 90.287 | 90.289 | 84.948 |
| LC10011b - D3 | 1051862_WT_0403 | 75.194 | 35 | 100 | 95 | 75.34 | 73.704 | 75.194 | 75.194 | 73.146 |
| LC10011b - D4 | 1051862_WT_0404 | 6.683 | 0 | 20 | 10 | 6.508 | 7.36 | 6.663 | 6.683 | 5.783 |
| LC10011b - D5 | 1051862_WT_0405 | 0.132 | 0 | 1 | <1 | 0 | 0.224 | 0.132 | 0.132 | 0.132 |
| LC10011b - D6 | 1051862_WT_0408 | 9.819 | 3 | 40 | 30 | 8.96 | 9.508 | 9.832 | 9.819 | 7.946 |
| LC10011b - D7 | 1051862_WT_0409 | 85.753 | 70 | 95 | 70 | 85.969 | 87.496 | 85.753 | 85.753 | 69.471 |
| LC10011b - D8 | 1051862_WT_0410 | 89.758 | 30 | 95 | 90 | 89.979 | 89.631 | 89.758 | 89.758 | 85.315 |
| LC10011b - D9 | 1051862_WT_0411 | 36.359 | 0 | 30 | 20 | 38.104 | 42.257 | 36.377 | 36.359 | 12.464 |
| LC10011b - D10 | 1051862_WT_0412 | 0.769 | 0 | 1 | 0 | 0.873 | 0.921 | 0.769 | 0.769 | 0.769 |
| LC10011b - E1 | 1051862_WT_0501 | 13.547 | 0 | 50 | 60 | 13.089 | 14.362 | 13.569 | 13.547 | 13.547 |
| LC10011b - E2 | 1051862_WT_0502 | 4.643 | 5 | 10 | 10 | 4.629 | 4.911 | 4.662 | 4.643 | 4.42 |
| LC10011b - E3 | 1051862_WT_0503 | 2.414 | 0 | 5 | 5 | 2.386 | 2.752 | 2.414 | 2.414 | 2.414 |
| LC10011b - E4 | 1051862_WT_0504 | 88.184 | 90 | 100 | 100 | 88.297 | 89.814 | 88.184 | 88.184 | 49.877 |
| LC10011b - E5 | 1051862_WT_0505 | 10.64 | 0 | 0 | <1 | 10.901 | 10.953 | 10.666 | 10.64 | 10.64 |
| LC10011b - E6 | 1051862_WT_0508 | 7.308 | 0 | 5 | 2 | 7.257 | 7.423 | 7.308 | 7.308 | 7.308 |
| LC10011b - E7 | 1051862_WT_0509 | 4.15 | 5 | 5 | 5 | 4.149 | 4.643 | 4.157 | 4.15 | 3.41 |
| LC10011b - E8 | 1051862_WT_0510 | 2.029 | 0 | 5 | 10 | 2.368 | 1.782 | 2.029 | 2.029 | 2.029 |
| LC10011b - E9 | 1051862_WT_0511 | 67.403 | 85 | 100 | 100 | 68.482 | 71.187 | 67.403 | 67.403 | 31.615 |
| LC10011b - E10 | 1051862_WT_0512 | 15.377 | 0 | 0 | 0 | 15.625 | 15.55 | 15.408 | 15.377 | 15.377 |
| LC10011b - F1 | 1051862_WT_0601 | 46.997 | 50 | 75 | 80 | 47.029 | 45.994 | 46.997 | 46.997 | 31.181 |
| LC10011b - F2 | 1051862_WT_0602 | 87.37 | 80 | 100 | 90 | 87.523 | 90.18 | 87.37 | 87.37 | 84.977 |
| LC10011b - F3 | 1051862_WT_0603 | 29.496 | 0 | 10 | 10 | 29.906 | 31.25 | 29.525 | 29.496 | 29.266 |
| LC10011b - F4 | 1051862_WT_0604 | 3.432 | 0 | 1 | 0 | 3.617 | 3.802 | 3.432 | 3.432 | 3.432 |
| LC10011b - F5 | 1051862_WT_0605 | 2.199 | 0 | 5 | <1 | 2.292 | 2.185 | 2.199 | 2.199 | 2.199 |
| LC10011b - F6 | 1051862_WT_0608 | 29.581 | 15 | 50 | 70 | 29.383 | 30.868 | 29.581 | 29.581 | 24.684 |
| LC10011b - F7 | 1051862_WT_0609 | 87.865 | 95 | 100 | 100 | 88.091 | 89.587 | 87.865 | 87.865 | 90.525 |
| LC10011b - F8 | 1051862_WT_0610 | 19.525 | 0 | 10 | 5 | 20.397 | 19.619 | 19.525 | 19.525 | 18.895 |
| LC10011b - F9 | 1051862_WT_0611 | 2.072 | 0 | 1 | 0 | 2.326 | 3.419 | 2.072 | 2.072 | 2.072 |
| LC10011b - F10 | 1051862_WT_0612 | 4.06 | 3 | 0 | 0 | 3.915 | 4.035 | 4.06 | 4.06 | 4 |
| LC10011b - G1 | 1051862_WT_0701 | 3.638 | 0 | 25 | 15 | 4.251 | 4.158 | 3.638 | 3.638 | 3.443 |
| LC10011b - G2 | 1051862_WT_0702 | 9.677 | NA | 1 | 1 | 11.538 | 7.143 | 9.677 | 9.677 | 9.677 |
| LC10011b - G3 | 1051862_WT_0703 | 27.992 | 0 | 0 | 0 | 29.877 | 37.405 | 28.006 | 27.992 | 27.992 |
| LC10011b - G4 | 1051862_WT_0704 | 3.475 | 0 | 10 | 5 | 3.216 | 3.937 | 3.475 | 3.475 | 3.272 |
| LC10011b - G5 | 1051862_WT_0705 | 5.066 | NA | 0 | 0 | 5.052 | 5.842 | 5.066 | 5.066 | 4.512 |
| LC10011b - G6 | 1051862_WT_0708 | 2.498 | 0 | 5 | 2 | 2.696 | 2.911 | 2.5 | 2.498 | 2.499 |
| LC10011b - G7 | 1051862_WT_0709 | 28 | NA | 0 | 0 | 27.941 | 29.167 | 28 | 28 | 28 |
| LC10011b - G8 | 1051862_WT_0710 | 4.211 | NA | 0 | 0 | 3.727 | 6.087 | 4.211 | 4.211 | 4.211 |
| LC10011b - G9 | 1051862_WT_0711 | 1.208 | 0 | <1 | <1 | 1.276 | 1.421 | 1.208 | 1.208 | 1.208 |
| LC10011b - G10 | 1051862_WT_0712 | 44.402 | 4 | 30 | 40 | 45.967 | 50.476 | 44.375 | 44.402 | 43.845 |
| LC10011b - H1 | 1051862_WT_0801 | 29.296 | 0 | 30 | 40 | 28.414 | 27.086 | 29.287 | 29.296 | 22.099 |
| LC10011b - H2 | 1051862_WT_0802 | 0.273 | 0 | 0 | <1 | 0.271 | 0.266 | 0.273 | 0.273 | 0.273 |
| LC10011b - H3 | 1051862_WT_0803 | 1.893 | 0 | 1 | <1 | 2.042 | 1.685 | 1.893 | 1.893 | 1.823 |
| LC10011b - H4 | 1051862_WT_0804 | 2.804 | 0 | 0 | 0 | 2.632 | 3.063 | 2.804 | 2.804 | 2.804 |
| LC10011b - H5 | 1051862_WT_0805 | 16.37 | 0 | 5 | 2 | 16.457 | 15.023 | 16.37 | 16.37 | 16.343 |
| LC10011b - H6 | 1051862_WT_0808 | 3.863 | 0 | 10 | 5 | 3.903 | 3.891 | 3.865 | 3.863 | 3.863 |
| LC10011b - H7 | 1051862_WT_0809 | 0.219 | 0 | 0 | 0 | 0.261 | 0.275 | 0.219 | 0.219 | 0.195 |
| LC10011b - H8 | 1051862_WT_0810 | 0.256 | 0 | 0 | 0 | 0.349 | 0.328 | 0.256 | 0.256 | 0.256 |
| LC10011b - H9 | 1051862_WT_0811 | 10.842 | 0 | 1 | <1 | 9.926 | 10.989 | 10.842 | 10.842 | 7.439 |
| LC10011b - H10 | 1051862_WT_0812 | 21.81 | 0 | 0 | 0 | 22.173 | 19.403 | 21.81 | 21.81 | 21.81 |

**Table S3.** Stain-Identified and Predicted Macrophage Counts.

| File name | Brightfield Cell Count | Predicted Macrophage Counts | Fluorescent Cell Count | CD-163 Positive Cell Count | CD-68 Positive Cell Count | Stain-Identified Macrophages |
| --- | --- | --- | --- | --- | --- | --- |
| Core_102 | 3020 | 101 | 3263 | 17 | 33 | 47 |
| Core_103 | 3048 | 0 | 2883 | 5 | 2 | 7 |
| Core_104 | 1145 | 0 | 954 | 0 | 1 | 1 |
| Core_105 | 8114 | 1370 | 5965 | 99 | 4 | 101 |
| Core_108 | 1730 | 0 | 399 | 74 | 2 | 75 |
| Core_109 | 3592 | 1 | 3379 | 5 | 2 | 7 |
| Core_110 | 5625 | 0 | 4172 | 12 | 0 | 12 |
| Core_111 | 801 | 0 | 787 | 2 | 0 | 2 |
| Core_112 | 6664 | 115 | 5326 | 183 | 25 | 195 |
| Core_201 | 6410 | 63 | 6482 | 66 | 32 | 96 |
| Core_202 | 3717 | 369 | 4033 | 249 | 327 | 438 |
| Core_203 | 5441 | 164 | 5233 | 54 | 132 | 152 |
| Core_204 | 6872 | 0 | 6105 | 75 | 9 | 81 |
| Core_205 | 4446 | 421 | 4347 | 50 | 11 | 58 |
| Core_208 | 6130 | 242 | 5444 | 12 | 16 | 28 |
| Core_209 | 5140 | 94 | 4489 | 84 | 147 | 193 |
| Core_210 | 3975 | 51 | 3780 | 60 | 108 | 148 |
| Core_211 | 8374 | 0 | 6330 | 106 | 17 | 114 |
| Core_212 | 3817 | 81 | 3959 | 40 | 20 | 56 |
| Core_301 | 4694 | 112 | 4405 | 7 | 8 | 14 |
| Core_303 | 2025 | 0 | 2844 | 64 | 25 | 79 |
| Core_304 | 3118 | 217 | 3594 | 23 | 17 | 38 |
| Core_305 | 5348 | 18 | 5181 | 200 | 143 | 290 |
| Core_308 | 6114 | 27 | 5357 | 47 | 11 | 52 |
| Core_309 | 4886 | 99 | 4656 | 288 | 165 | 323 |
| Core_310 | 2094 | 62 | 2449 | 191 | 11 | 198 |
| Core_311 | 3327 | 236 | 3872 | 113 | 12 | 118 |
| Core_312 | 6627 | 21 | 4948 | 76 | 62 | 117 |
| Core_402 | 6521 | 1110 | 5575 | 82 | 79 | 135 |
| Core_404 | 6234 | 710 | 5757 | 136 | 113 | 212 |
| Core_405 | 1548 | 11 | 3131 | 23 | 3 | 26 |
| Core_409 | 6320 | 1057 | 4246 | 26 | 25 | 45 |
| Core_410 | 1828 | 97 | 2240 | 9 | 1 | 10 |
| Core_411 | 4510 | 1097 | 5196 | 433 | 628 | 811 |
| Core_412 | 3430 | 0 | 4093 | 38 | 8 | 44 |
| Core_501 | 4052 | 38 | 4313 | 140 | 11 | 144 |
| Core_502 | 5176 | 103 | 5025 | 0 | 1 | 1 |
| Core_503 | 4873 | 0 | 4036 | 139 | 4 | 140 |
| Core_504 | 5023 | 1690 | 4654 | 190 | 75 | 242 |
| Core_505 | 4309 | 0 | 5211 | 12 | 56 | 66 |
| Core_508 | 3202 | 0 | 3634 | 49 | 7 | 55 |
| Core_509 | 5577 | 0 | 4769 | 1 | 0 | 1 |
| Core_510 | 5994 | 0 | 4617 | 256 | 31 | 269 |
| Core_511 | 3582 | 392 | 4532 | 71 | 47 | 115 |
| Core_512 | 6662 | 0 | 4611 | 10 | 32 | 42 |
| Core_601 | 4062 | 886 | 4683 | 305 | 88 | 329 |
| Core_602 | 3442 | 1600 | 3390 | 234 | 239 | 350 |
| Core_603 | 2809 | 117 | 3979 | 46 | 204 | 223 |
| Core_604 | 3671 | 0 | 4377 | 4 | 1 | 5 |
| Core_605 | 4844 | 24 | 3883 | 66 | 71 | 129 |
| Core_608 | 4069 | 699 | 4080 | 372 | 501 | 676 |
| Core_609 | 2758 | 919 | 3316 | 289 | 272 | 415 |
| Core_610 | 4843 | 53 | 4844 | 305 | 241 | 419 |
| Core_611 | 2231 | 0 | 3276 | 3 | 0 | 3 |
| Core_612 | 4556 | 8 | 3505 | 36 | 42 | 73 |
| Core_701 | 4173 | 7 | 3845 | 7 | 10 | 17 |
| Core_702 | 988 | 0 | 1989 | 93 | 12 | 99 |
| Core_703 | 3092 | 0 | 3562 | 5 | 8 | 12 |
| Core_704 | 2547 | 1 | 2863 | 2 | 11 | 13 |
| Core_705 | 2457 | 7 | 3662 | 4 | 3 | 7 |
| Core_708 | 3589 | 9 | 3836 | 26 | 60 | 83 |
| Core_711 | 1915 | 0 | 2636 | 5 | 17 | 22 |
| Core_712 | 4355 | 39 | 4087 | 111 | 34 | 140 |
| Core_801 | 5571 | 59 | 4757 | 1 | 12 | 13 |
| Core_802 | 6199 | 0 | 4619 | 6 | 17 | 19 |
| Core_803 | 3686 | 12 | 3777 | 34 | 255 | 269 |
| Core_804 | 4467 | 22 | 4402 | 49 | 126 | 159 |
| Core_805 | 3485 | 2 | 639 | 0 | 0 | 0 |
| Core_808 | 6129 | 0 | 5100 | 15 | 4 | 16 |
| Core_809 | 5482 | 4 | 4508 | 39 | 34 | 67 |
| Core_810 | 4856 | 1 | 4202 | 8 | 124 | 130 |
| Core_811 | 4845 | 84 | 5061 | 159 | 98 | 233 |
| Core_812 | 2348 | 1 | 394 | 0 | 0 | 0 |
| Lung_Ad_19 | 509092 | 188886 | 689961 | 109898 | 13225 | 113294 |
| Lung_Ad_27 | 773177 | 276097 | 1118390 | 62343 | 34768 | 83718 |
| Lung_Ad_29 | N/A | N/A | 134196 | 12124 | 2838 | 13824 |
| Lung_SCC_4 | 73864 | 22581 | 62163 | 1927 | 1633 | 3534 |
| NSCLC_143 | 366826 | 35427 | 298477 | 23981 | 4366 | 25406 |
| NSCLC_93-1 | 181907 | 27700 | 149997 | 1728 | 1068 | 2623 |
| NSCLC_93 | 22670 | 9438 | 40015 | 6422 | 832 | 6786 |
| NSCLC_95-2 | 193272 | 63605 | 227601 | 23532 | 10396 | 31820 |
| NSCLC_98-2 | 4393 | 56 | 7521 | 134 | 140 | 264 |
| NSCLC_146 | 733696 | 173012 | 919201 | 24385 | 3946 | 27417 |
| NSCLC_147 | 817037 | 98944 | 1028710 | 69285 | 19999 | 77284 |
| NSCLC_148 | 727795 | 46254 | 622967 | 4448 | 884 | 5330 |
| NSCLC_149 | 735108 | 108930 | 852778 | 67402 | 11580 | 74163 |
| NSCLC_150 | 685322 | 44158 | 574395 | 96874 | 5400 | 98237 |
| NSCLC_151 | 573582 | 180017 | 879244 | 281449 | 40059 | 298135 |
| NSCLC_152 | 396027 | 56805 | 466094 | 42329 | 24609 | 62199 |
| NSCLC_153 | 1700840 | 18404 | 1191080 | 13764 | 4141 | 16709 |
| NSCLC_154 | 1160450 | 155379 | 864566 | 240143 | 4930 | 243964 |
| NSCLC_155 | 936785 | 214856 | 1065450 | 68296 | 44255 | 102684 |
| NSCLC_156 | 699738 | 92581 | 1172390 | 14923 | 27250 | 37378 |
| NSCLC_157 | 644315 | 58721 | 621044 | 9963 | 2395 | 11783 |
| NSCLC_158 | 1107960 | 138370 | 1455980 | 25543 | 14923 | 37973 |
| NSCLC_159 | 1355340 | 241456 | 956171 | 34367 | 9246 | 40012 |
| NSCLC_160 | 94724 | 17480 | 92447 | 2839 | 2158 | 4238 |
| NSCLC_161 | 634459 | 134734 | N/A | N/A | N/A | N/A |
| NSCLC_162 | 516174 | 68266 | N/A | N/A | N/A | N/A |
| NSCLC_163 | 621273 | 136638 | 785287 | 167376 | 59355 | 191661 |

**Table S4.** Stain-Identified and Predicted Lymphocyte Counts

| File name | Brightfield Total Cell Count | Predicted Lymphocyte Count | Percent Predicted Lymphocytes | IF Total Cell Count | Total IF Lymphocyte Count | Percent-Stained Lymphocytes | CD3+ Cell Count | CD20+ Cell Count |
| --- | --- | --- | --- | --- | --- | --- | --- | --- |
| Core_102 | 3020 | 1 | 0.03 | 3594 | 102 | 2.84 | 101 | 1 |
| Core_103 | 3048 | 1 | 0.03 | 3450 | 18 | 0.52 | 18 | 0 |
| Core_104 | 1145 | 1 | 0.09 | 1859 | 2 | 0.11 | 2 | 0 |
| Core_105 | 8114 | 22 | 0.27 | 6639 | 2 | 0.03 | 2 | 0 |
| Core_108 | 1730 | 3 | 0.17 | 1035 | 0 | 0 | 0 | 0 |
| Core_109 | 3592 | 18 | 0.5 | 4285 | 105 | 2.45 | 104 | 1 |
| Core_110 | 5625 | 0 | 0 | 4766 | 0 | 0 | 0 | 0 |
| Core_111 | 801 | 0 | 0 | 588 | 1 | 0.17 | 1 | 0 |
| Core_112 | 6664 | 12 | 0.18 | 6407 | 7 | 0.11 | 7 | 0 |
| Core_201 | 6410 | 141 | 2.2 | 6658 | 130 | 1.95 | 112 | 19 |
| Core_202 | 3717 | 114 | 3.07 | 4115 | 55 | 1.34 | 55 | 0 |
| Core_203 | 5441 | 26 | 0.48 | 6133 | 389 | 6.34 | 374 | 15 |
| Core_204 | 6872 | 87 | 1.27 | 7134 | 15 | 0.21 | 3 | 12 |
| Core_205 | 4446 | 11 | 0.25 | 4946 | 3 | 0.06 | 3 | 0 |
| Core_208 | 6130 | 207 | 3.38 | 6069 | 13 | 0.21 | 13 | 0 |
| Core_209 | 5140 | 45 | 0.88 | 5300 | 88 | 1.66 | 88 | 0 |
| Core_210 | 3975 | 31 | 0.78 | 4488 | 36 | 0.8 | 36 | 0 |
| Core_211 | 8374 | 203 | 2.42 | 8005 | 5 | 0.06 | 4 | 1 |
| Core_212 | 3817 | 6 | 0.16 | 4809 | 3 | 0.06 | 2 | 1 |
| Core_301 | 4694 | 15 | 0.32 | 5598 | 1 | 0.02 | 1 | 0 |
| Core_303 | 2025 | 2 | 0.1 | 2790 | 2 | 0.07 | 2 | 0 |
| Core_304 | 3118 | 10 | 0.32 | 3906 | 15 | 0.38 | 15 | 0 |
| Core_305 | 5348 | 12 | 0.22 | 5619 | 101 | 1.8 | 98 | 3 |
| Core_308 | 6114 | 29 | 0.47 | 6113 | 3 | 0.05 | 3 | 0 |
| Core_309 | 4886 | 13 | 0.27 | 5558 | 134 | 2.41 | 128 | 7 |
| Core_310 | 2094 | 0 | 0 | 2484 | 17 | 0.68 | 17 | 0 |
| Core_311 | 3327 | 17 | 0.51 | 4375 | 9 | 0.21 | 9 | 0 |
| Core_312 | 6627 | 17 | 0.26 | 5884 | 197 | 3.35 | 113 | 88 |
| Core_402 | 6521 | 47 | 0.72 | 6099 | 663 | 10.87 | 637 | 33 |
| Core_404 | 6234 | 63 | 1.01 | 6298 | 160 | 2.54 | 127 | 33 |
| Core_405 | 1548 | 1 | 0.06 | 3955 | 0 | 0 | 0 | 0 |
| Core_409 | 6320 | 107 | 1.69 | 5564 | 399 | 7.17 | 396 | 3 |
| Core_410 | 1828 | 3 | 0.16 | 2849 | 26 | 0.91 | 26 | 0 |
| Core_411 | 4510 | 21 | 0.47 | 4972 | 128 | 2.57 | 125 | 3 |
| Core_412 | 3430 | 5 | 0.15 | 4830 | 0 | 0 | 0 | 0 |
| Core_501 | 4052 | 45 | 1.11 | 4617 | 17 | 0.37 | 0 | 17 |
| Core_502 | 5176 | 48 | 0.93 | 5276 | 14 | 0.27 | 14 | 0 |
| Core_503 | 4873 | 11 | 0.23 | 5238 | 10 | 0.19 | 10 | 0 |
| Core_504 | 5023 | 33 | 0.66 | 4990 | 0 | 0 | 0 | 0 |
| Core_505 | 4309 | 431 | 10 | 5871 | 25 | 0.43 | 20 | 5 |
| Core_508 | 3202 | 29 | 0.91 | 3924 | 81 | 2.06 | 81 | 0 |
| Core_509 | 5577 | 10 | 0.18 | 5094 | 33 | 0.65 | 33 | 0 |
| Core_510 | 5994 | 62 | 1.03 | 5645 | 50 | 0.89 | 33 | 17 |
| Core_511 | 3582 | 11 | 0.31 | 5116 | 0 | 0 | 0 | 0 |
| Core_512 | 6662 | 268 | 4.02 | 5240 | 67 | 1.28 | 22 | 45 |
| Core_601 | 4062 | 33 | 0.81 | 4779 | 89 | 1.86 | 89 | 0 |
| Core_602 | 3442 | 1 | 0.03 | 3342 | 216 | 6.46 | 214 | 2 |
| Core_603 | 2809 | 10 | 0.36 | 4749 | 760 | 16 | 740 | 21 |
| Core_604 | 3671 | 4 | 0.11 | 4660 | 4 | 0.09 | 3 | 1 |
| Core_605 | 4844 | 12 | 0.25 | 4138 | 2 | 0.05 | 0 | 2 |
| Core_608 | 4069 | 14 | 0.34 | 3995 | 517 | 12.94 | 517 | 0 |
| Core_609 | 2758 | 2 | 0.07 | 3371 | 522 | 15.49 | 520 | 2 |
| Core_610 | 4843 | 26 | 0.54 | 5467 | 367 | 6.71 | 357 | 10 |
| Core_611 | 2231 | 2 | 0.09 | 3524 | 0 | 0 | 0 | 0 |
| Core_612 | 4556 | 4 | 0.09 | 4120 | 2 | 0.05 | 0 | 2 |
| Core_701 | 4173 | 14 | 0.34 | 4161 | 0 | 0 | 0 | 0 |
| Core_702 | 988 | 0 | 0 | 1914 | 0 | 0 | 0 | 0 |
| Core_703 | 3092 | 2 | 0.06 | 4013 | 13 | 0.32 | 13 | 0 |
| Core_704 | 2547 | 0 | 0 | 2951 | 9 | 0.3 | 9 | 0 |
| Core_705 | 2457 | 56 | 2.28 | 4362 | 765 | 17.54 | 763 | 2 |
| Core_708 | 3589 | 7 | 0.2 | 3830 | 2 | 0.05 | 2 | 0 |
| Core_711 | 1915 | 0 | 0 | 2795 | 32 | 1.14 | 32 | 0 |
| Core_712 | 4355 | 5 | 0.11 | 4718 | 40 | 0.85 | 15 | 25 |
| Core_801 | 5571 | 1 | 0.02 | 5044 | 26 | 0.52 | 23 | 3 |
| Core_802 | 6199 | 7 | 0.11 | 4982 | 0 | 0 | 0 | 0 |
| Core_803 | 3686 | 12 | 0.33 | 3867 | 64 | 1.66 | 61 | 3 |
| Core_804 | 4467 | 11 | 0.25 | 4669 | 180 | 3.86 | 178 | 2 |
| Core_805 | 3485 | 0 | 0 | 620 | 3 | 0.48 | 3 | 0 |
| Core_808 | 6129 | 8 | 0.13 | 4975 | 85 | 1.71 | 85 | 0 |
| Core_809 | 5482 | 36 | 0.66 | 4761 | 0 | 0 | 0 | 0 |
| Core_810 | 4856 | 15 | 0.31 | 4436 | 21 | 0.47 | 21 | 0 |
| Core_811 | 4845 | 19 | 0.39 | 5415 | 450 | 8.31 | 447 | 3 |
| Core_812 | 2348 | 2 | 0.09 | 385 | 0 | 0 | 0 | 0 |
| Lung_Adeno_19 | 509092 | 30678 | 6.03 | 694533 | 54025 | 7.78 | 54019 | 8 |
| Lung_Adeno_27 | 773177 | 66746 | 8.63 | 1143836 | 209536 | 18.32 | 209451 | 106 |
| Lung_SCC_4 | 73864 | 20462 | 27.7 | 41529 | 5146 | 12.39 | 4066 | 1272 |
| NSCLC_143 | 366826 | 9058 | 2.47 | 273893 | 1261 | 0.46 | 1234 | 27 |
| NSCLC_146 | 733696 | 3725 | 0.51 | 764320 | 12176 | 1.59 | 12173 | 3 |
| NSCLC_147 | 817037 | 95880 | 11.74 | 978621 | 162271 | 16.58 | 162100 | 227 |
| NSCLC_148 | 727795 | 88783 | 12.2 | 594509 | 31394 | 5.28 | 30153 | 1372 |
| NSCLC_149 | 735108 | 57778 | 7.86 | 836198 | 98380 | 11.77 | 98294 | 100 |
| NSCLC_150 | 685322 | 21124 | 3.08 | 532128 | 9920 | 1.86 | 9917 | 3 |
| NSCLC_151 | 573582 | 8292 | 1.45 | 682464 | 83793 | 12.28 | 83485 | 410 |
| NSCLC_152 | 396027 | 67219 | 16.97 | 321685 | 84213 | 26.18 | 84131 | 96 |
| NSCLC_153 | 1700841 | 69626 | 4.09 | 1234634 | 77730 | 6.3 | 77043 | 707 |
| NSCLC_154 | 1160450 | 76060 | 6.55 | 810709 | 13855 | 1.71 | 13832 | 25 |
| NSCLC_155 | 936785 | 98258 | 10.49 | 926211 | 46420 | 5.01 | 46405 | 18 |
| NSCLC_156 | 699738 | 57152 | 8.17 | 1290763 | 83512 | 6.47 | 83222 | 292 |
| NSCLC_157 | 644315 | 112307 | 17.43 | 542177 | 26908 | 4.96 | 26844 | 65 |
| NSCLC_158 | 1107960 | 110396 | 9.96 | 1385218 | 65572 | 4.73 | 65345 | 234 |
| NSCLC_159 | 1355341 | 134549 | 9.93 | 739752 | 38863 | 5.25 | 38831 | 32 |
| NSCLC_160 | 94724 | 13591 | 14.35 | 94842 | 10310 | 10.87 | 10288 | 23 |
| NSCLC_161 | 634459 | 33379 | 5.26 | 709423 | 33848 | 4.77 | 33834 | 14 |
| NSCLC_162 | 516174 | 76771 | 14.87 | 599530 | 49101 | 8.19 | 49080 | 23 |
| NSCLC_163 | 621273 | 54239 | 8.73 | 720976 | 50433 | 7 | 49986 | 475 |
| NSCLC_93_1 | 181907 | 17910 | 9.85 | 139470 | 4654 | 3.34 | 4648 | 6 |
| NSCLC_93 | 22670 | 2443 | 10.78 | 35606 | 3567 | 10.02 | 3561 | 7 |
| NSCLC_95_2 | 193272 | 42283 | 21.88 | 195153 | 30426 | 15.59 | 30416 | 11 |
| NSCLC_98_2 | 4393 | 69 | 1.57 | 6541 | 440 | 6.73 | 440 | 0 |
